# Assessment of interaction between maternal polycyclic aromatic hydrocarbons exposure and genetic polymorphisms on the risk of congenital heart diseases

**DOI:** 10.1038/s41598-018-21380-3

**Published:** 2018-02-15

**Authors:** Nana Li, Yi Mu, Zhen Liu, Ying Deng, Yixiong Guo, Xuejuan Zhang, Xiaohong Li, Ping Yu, Yanping Wang, Jun Zhu

**Affiliations:** 1 National Center for Birth Defect Monitoring, West China Second University Hospital, Sichuan University, Sec.3 No. 17, South RenMin Road, Chengdu, Sichuan China; 2 Key Laboratory of Birth Defects and Related Diseases of Women and Children (Sichuan University), Ministry of Education, Chengdu, Sichuan China; 3 Women Health Care Department, Shanxi Women and Children Health Center, Children’s Hospital of Shanxi, Taiyuan, Shanxi, China

## Abstract

The major causes of congenital heart diseases (CHDs) are the interactions of genetic and environmental factors. We conducted a case–control study in 357 mothers of CHDs fetuses and 270 control mothers to investigate the association of maternal PAHs exposure, AHR, CYP1A1, CYP1A2, CYP1B1 and CYP2E polymorphisms, the interaction between PAHs exposure and genetic variants with the risk of CHDs. The higher level PAHs exposure was associated with the risk of CHDs (aOR = 2.029, 95% CI: 1.266, 3.251) or subtypes. The haplotypes of AHR or CYP1A2 were associated with the risk of CHDs: AHR: C-G-A-C: aOR = 0.765; T-A-G-A: aOR = 1.33; CYP1A2: A-T:aOR = 1.75; C-C: aOR = 0.706. When exposed to higher level PAHs, the risk of CHDs among the mothers carrying rs2158041 “C/T or T/T” genotype or rs7811989 “G/A or A/A” genotype in AHR was 1.724 (χ^2^ = 7.209, P = 0.007) or 1.735 (χ^2^ = 7.364, P = 0.007) times greater than the aOR in the mothers carrying wild genotype. The multiplicative-scale interactions between PAHs exposure and polymorphisms of CYP1A2 rs4646425 (P = 0.03) or CYP2E1 rs915908 (P = 0.0238) on the risk of CHDs were observed. Our study suggests that maternal AHR polymorphisms may modify the association of PAHs exposure with CHDs, CYP1A2 or CYP2E1 polymorphisms significantly interact with PAHs exposure on CHDs.

## Introduction

Congenital heart diseases (CHDs) are the most common type of birth defect, accounting for one-third of all major congenital anomalies^1^. Approximately, 4~10 of live births are affected by CHDs^2,3^. In China, the average total prevalence of CHDs was 40.95 per 10,000 live births in 2011, and 130 thousand infants are born with CHDs each year^4^. Consequently, CHDs cause considerable suffering to patients and their families, and CHDs have become a sizable public health concern.

Although advances in the understanding of the genetic risk factors and environmental risk factors affecting the development of CHDs have been made, the aetiology of the majority of CHDs remains unknown^5–7^. Now, it is widely believed that most CHDs arise from a complex and ill-defined combination of genetic and environmental factors^8,9^.

Polycyclic aromatic hydrocarbons (PAHs) are widespread environmental pollutants formed by the incomplete burning of coal, tobacco, or other organic substances^10^. The general population is unavoidably exposed to PAHs through the inhalation of tobacco smoke, smoke from other sources of combustion, and ambient air and through the consumption of PAHs, particularly PAHs in charbroiled foods^11^. PAHs have been found in placental tissues^12^ and umbilical cord blood^12^, which suggests that transplacental transfer of these chemicals to the foetus can have a significant impact on foetal development, including increased risk of neural tube defects, cleft lip with or without cleft palate, gastroschisis, low birth weight, preterm birth, and intrauterine growth restriction^13–18^. Although studies of experimental model systems have suggested that prenatal exposure to PAHs is associated with CHDs^19,20^, the results of a large, population-based study did not support the association between potential maternal occupational exposure to PAHs and CHDs^21^. Owing to the limitation of using expert industrial hygienists’ assessments of exposure to PAHs, further evidence and quantitative data are needed to illustrate the association between prenatal exposure to PAHs and CHDs.

The cytochrome P450 (CYP) enzymes CYP1A1, CYP1A2, and CYP1B1 have been shown to play important roles in the metabolic activation of PAHs^22^. CYP2E1 is expressed at higher levels in Asians than in Caucasians; therefore, it is thought to be responsible for the metabolism of pyrene in Asian people^23^. The aryl hydrocarbon receptor (AHR), a ligand-activated transcription factor, mediates the toxic effects of a variety of environmental chemicals, including PAHs^24^, as well as the induction of three members of the CYP1 family, CYP1A1, CYP1A2 and CYP1B1^25^. Common genetic polymorphisms in these genes could affect individual susceptibility to adverse effects of exposure to PAHs. Maternal genotypes, such as those involving the gene CYP1B1, have been shown to enhance the association between maternal exposure to PAHs and neural tube defects^26^. However, few studies have investigated maternal genetic susceptibility to CHDs related to PAHs or have explored possible gene-environment interactions.

1-Hydroxypyrene-glucuronide (1-OHPG) is a stable PAH metabolite that is excreted in the urine and is an index biomarker that reflects recent exposure to mixed PAHs^27^. In the present study, we first analysed the association between maternal exposure to PAHs by measuring urine 1-OHPG concentration during pregnancy and the risk of foetal CHDs. Then, we investigated the association between maternal genetic polymorphisms and the risk of foetal CHDs. Finally, we evaluated the potential interaction between maternal genetic variants and exposure to PAHs on the risk of foetal CHDs.

## Results

### Characteristics of the study participants

In this study, a total of 627 participants were analysed (357 cases and 270 controls). The baseline characteristics of the participants are presented in Table 1. There were significant differences between the two groups with respect to gestational week, cooking at home, maternal alcohol consumption, and folic acid supplements.

**Table 1:** Characteristics of the case and control participants.

Variables/Characteristic	Cases	Controls	*χ* ^2^	*P*-values
	No.(%)	No.(%)		
Maternal age (yrs)^a^			4.096	0.129
<25	99(27.7)	58(21.5)		
25–34	211(59.1)	166(61.5)		
≥35	47(13.2)	46(17.0)		
Gestational week^a^			103.038	<0.001
<19	13(3.6)	88(32.6)		
20–25	194(54.3)	126(46.7)		
26–31	109(30.5)	42(15.6)		
≥32	41(11.5)	14(5.2)		
Housing renovation^b^			0.766	0.381
Yes	79(22.1)	52(19.3)		
No	278(77.9)	218(80.7)		
Factory or landfill nearby^b^			0.460	0.498
Yes	56(15.7)	37(13.7)		
No	298(83.5)	230(85.2)		
Cooking at home^b^			13.021	0.001
Often	203(56.9)	122(45.2)		
Never	76(21.3)	91(33.7)		
Occasional	75(21.0)	54(20.0)		
Parental smoking or ETS exposure^b^			1.758	0.185
Yes	223(62.5)	155(57.4)		
No	133(37.3)	115(42.6)		
Maternal alcohol consumption^b^			10.671	0.005
Often	6(1.7)	8(3.0)		
Occasional	55(15.4)	68(25.2)		
Never	293(82.1)	194(71.9)		
Folic acid supplements^b^			4.057	0.044
Yes	297(83.2)	240(88.9)		
No	60(16.8)	30(11.1)		

^a^Using base data in following multivariate analysis as continuous variables.

^b^The exposure was defined from the 3 months before pregnancy to the first trimester.

### Association between maternal exposure to PAHs and the risk of CHDs

Table 2 displays the relation between maternal exposure to PAHs and foetal CHDs. Significant positive associations were observed between maternal exposure to PAHs and various CHDs phenotypes when comparing high exposure to low exposure after adjusting for potential confounders: all CHDs (aOR = 2.029; 95% CI: 1.266, 3.251), septal defects (aOR = 2.373, 95% CI: 1.376, 4.093), conotruncal heart defects (aOR = 2.349, 95% CI: 1.250, 4.416), right-sided obstructive malformations (aOR = 2.423, 95% CI: 1.190, 4.933), left-sided obstructive malformations (aOR = 2.662, 95% CI: 1.085, 6.529), anomalous pulmonary venous return (aOR = 2.962, 95% CI: 1.068, 8.212), and other heart abnormalities (aOR = 2.327, 95% CI: 1.129, 4.795).

**Table 2:** Association between maternal PAHs exposure and the risk of CHDs.

PAHs exposure	Cases	Controls	cOR(95%CI)	aOR^b^(95%CI)
	No.(%)	No.(%)		
Any CHDs				
low	43(12.0)	62(23.0)	Ref.	Ref.
high	314(88.0)	208(77.0)	2.177(1.421,3.335)	2.029(1.266,3.251)*
septal defects				
low	27(11.5)	62(23.0)	Ref.	Ref.
high	208(88.5)	208(77.0)	2.296(1.405,3.752)	2.373(1.376,4.093)*
conotruncal heart defects				
low	16(10.0)	62(23.0)	Ref.	Ref.
high	144(90.0)	208(77.0)	2.683(1.488,4.836)	2.349(1.250,4.416)*
right-sided obstructive malformations				
low	11(9.6)	62(23.0)	Ref.	Ref.
high	103(90.4)	208(77.0)	2.791(1.409,5.528)	2.423(1.190,4.933)*
left-sided obstructive malformations				
low	7(9.7)	62(23.0)	Ref.	Ref.
high	65(90.3)	208(77.0)	2.768(1.207,6.345)	2.662(1.085,6.529)*
anomalous pulmonary venous return				
low	5(7.8)	62(23.0)	Ref.	Ref.
high	59(92.2)	208(77.0)	3.517(1.352,9.149)	2.962(1.068,8.212)*
other cardiac structural abnormalities				
low	11(10.8)	62(23.0)	Ref.	Ref.
high	91(89.2)	208(77.0)	2.466(1.241,4.906)	2.327(1.129,4.795)*

cOR: crude odds ration; aOR: adjusted odds ration, adjusted for maternal age, gestational week, housing renovation, factory or landfill nearby, cooking at home, parental smoking or ETS exposure, maternal alcohol consumption, folic acid supplements.

### Association between maternal gene polymorphisms and the risk of CHDs

The genotype frequencies for polymorphisms of AHR, CYP1A1, CYP1A2, CYP1B1 and CYP2E1 in the controls were in Hardy-Weinberg equilibrium (see Supplementary Appendix A, Table S1).

Table 3 shows the association between single gene loci polymorphisms and the risk of CHDs, assuming various genetic models. In the AHR gene, the SNPs rs2158041 and rs7811989 were associated with an increased risk of CHDs under the dominant model (aOR = 1.454, 95% CI: 1.024, 2.065; aOR = 1.46, 95% CI: 1.027, 2.075), and the SNPs rs2066853 and rs2040623 were associated with a decreased risk of CHDs under the additive model (aOR = 0.7648, 95% CI: 0.5859, 0.9983; aOR = 0.761, 95% CI: 0.5867, 0.9872). In the CYP1A2 gene, the SNP rs762551 was associated with a decreased risk of CHDs (under the dominant model: aOR = 0.6529, 95% CI: 0.4608, 0.9252; under the additive model: aOR = 0.7062, 95% CI: 0.5444, 0.916) and the SNP rs4646425 was associated with an increased risk of CHDs (under the dominant model: aOR = 1.723, 95% CI: 1.048, 2.833; under the additive model: aOR = 1.748, 95% CI: 1.077, 2.839). The SNPs rs2158041, rs7811989, rs762551 and rs4646425 were associated with some subtypes of CHDs; the data are shown in Supplementary Appendix B, Table S2. However, the associations were not statistically significant after the false discovery rate (FDR) correction. No significant association was found between any of the remaining 16 selected loci and the risk of CHDs or any CHDs subtype before or after the FDRcorrection.

**Table 3:** Association between maternal genotypes and the risk of CHDs.

dbSNP_ID	Model	Genotype	Controls	Cases	aOR(95%CI)	*P*-value	FDR-BH *P* value
			N(%)	N(%)			
rs2158041	Dominant	C/C	168 (62.2)	194 (54.3)	1	**0.03656**	**0.1828**
		C/T- T/T	102 (37.8)	163 (45.7)	1.454 (1.024, 2.065)*		
	Recessive	C/C- C/T	253 (93.7)	331 (92.7)	1	0.6361	0.8959
		T/T	17 (6.3)	26 (7.3)	1.179 (0.596, 2.332)		
	Log-additive	—	—	—	1.303 (0.9841, 1.725)	0.06457	0.2544
rs7811989	Dominant	G/G	169 (62.6)	194 (54.3)	1	**0.03491**	**0.1828**
		G/A-A/A	101 (37.4)	163 (45.7)	1.46 (1.027, 2.075)*		
	Recessive	G/G-G/A	252 (93.3)	331 (92.7)	1	0.7877	0.8959
		A/A	18 (6.7)	26 (7.3)	1.097 (0.5606, 2.145)		
	Log-additive	—	—	—	1.287 (0.9736, 1.702)	0.07631	0.2544
rs2066853	Dominant	G/G	111 (41.1)	163 (45.7)	1	0.1473	0.453
		G/A-A/A	159 (58.9)	194 (54.3)	0.7704 (0.5414,1.096)		
	Recessive	G/G-G/A	237 (87.8)	326 (91.3)	1	0.0622	0.4806
		A/A	33 (12.2)	31 (8.7)	0.5861 (0.3342,1.028)		
	Log-additive	—	—	—	0.7648 (0.5859,0.9983)*	**0.04855**	**0.2428**
rs2040623	Dominant	A/A	96 (35.6)	141 (39.5)	1	0.1137	0.453
		A/C-C/C	174 (64.4)	216 (60.5)	0.7453 (0.5178,1.073)		
	Recessive	A/A-A/C	228 (84.4)	314 (88.0)	1	0.07209	0.4806
		CC	42 (15.6)	43 (12.0)	0.6322 (0.3836,1.042)		
	Log-additive	—	—	—	0.761 (0.5867,0.9872)*	**0.03969**	**0.2428**
rs1048943	Dominant	T/T	146 (54.1)	194 (54.3)	1	0.9835	0.9835
		T/C- C/C	124 (45.9)	163 (45.7)	1.004 (0.7102, 1.418)		
	Recessive	T/T- T/C	254 (94.1)	330 (92.4)	1	0.3767	0.8126
		C/C	16 (5.9)	27 (7.6)	1.375 (0.6786, 2.786)		
	Log-additive	—	—	—	1.055 (0.7976, 1.395)	0.708	0.885
rs4646422	Dominant	C/C	201 (74.4)	284 (79.6)	1	0.1585	0.453
		C/T-T/T	69 (25.6)	73 (20.4)	0.7448 (0.4944, 1.122)		
	Recessive	C/C-C/T	268 (99.3)	351 (98.3)	1	0.439	0.8126
		T/T	2 (0.7)	6 (1.7)	1.934 (0.364, 10.27)		
	Log-additive	—	—	—	0.8108 (0.5575, 1.179)	0.2725	0.545
rs4642421	Dominant	G/G	84 (31.1)	108 (30.3)	1	0.6549	0.9835
		G/A-A/A	186 (68.9)	249 (69.7)	1.089 (0.7487,1.585)		
	Recessive	G/G-G/A	225 (83.3)	290 (81.2)	1	0.546	0.8401
		A/A	45 (16.7)	67 (18.8)	1.151 (0.7289,1.818)		
	Log-additive	—	—	—	1.087 (0.8425,1.402)	0.5219	0.8718
rs762551	Dominant	A/A	119 (44.1)	187 (52.4)	1	0.01651	0.1828
		A/C- C/C	151 (55.9)	170 (47.6)	0.6529 (0.4608, 0.9252)*		
	Recessive	A/A- A/C	235 (87.0)	328 (91.9)	1	0.0703	0.4806
		C/C	35 (13.0)	29 (8.1)	0.5969 (0.3414, 1.044)		
	Log-additive	—	—	—	**0.7062** (0.5444, 0.916)*	**0.008771**	**0.1754**
rs4646425	Dominant	C/C	238 (88.1)	291 (81.5)	1	**0.03186**	0.1828
		C/T-T/T	32 (11.9)	66 (18.5)	**1.723** (1.048, 2.833)*		
	Recessive	C/C-C/T	270 (100.0)	354 (99.2)	1	0.9988	0.9988
		T/T	0 (0.0)	3 (0.8)	—		
	Log-additive	—	—	—	1.748 (1.077, 2.839)*	**0.02394**	**0.2394**
rs2472304	Dominant	G/G	189 (70.0)	244 (68.3)	1	0.206	0.4578
		G/A-A/A	81 (30.0)	113 (31.7)	1.278 (0.874,1.867)		
	Recessive	G/G-G/A	265 (98.1)	349 (97.8)	1	0.7375	0.8959
		A/A	5 (1.9)	8 (2.2)	1.231 (0.3656,4.143)		
	Log-additive	—	—	—	1.241 (0.8806,1.748)	0.2175	0.5438
rs2470890	Dominant	G/G	189 (70.0)	244 (68.3)	1	0.1935	0.4578
		G/A-A/A	81 (30.0)	113 (31.7)	1.287 (0.88,1.881)		
	Recessive	G/G-G/A	265 (98.1)	349 (97.8)	1	0.7375	0.8959
		A/A	5 (1.9)	8 (2.2)	1.231 (0.3656,4.143)		
	Log-additive	—	—	—	1.248 (0.8855,1.758)	0.2058	0.5438
rs2855658	Dominant	C/C	217 (80.4)	283 (79.3)	1	0.9736	0.9835
		C/T- T/T	53 (19.6)	74 (20.7)	1.007 (0.6578, 1.542)		
	Recessive	C/C - C/T	265 (98.1)	353 (98.9)	1	0.9292	0.9781
		T/T	5 (1.9)	4 (1.1)	0.9364 (0.2199, 3.987)		
	Log-additive	—	—	—	1.001 (0.6818, 1.47)	0.995	0.995
rs1056837	Dominant	G/G	217 (80.4)	282 (79.0)	1	0.8692	0.9835
		G/A-A/A	53 (19.6)	75 (21.0)	1.036 (0.6772, 1.586)		
	Recessive	G/G-G/A	265 (98.1)	353 (98.9)	1	0.4875	0.8126
		A/A	6 (1.9)	4 (1.1)	0.6042 (0.1457, 2.506)		
	Log-additive	—	—	—	0.9918 (0.6774, 1.452)	0.9663	0.995
rs1056836	Dominant	G/G	216 (80.0)	280 (78.4)	1	0.8848	0.9835
		G/C- C/C	54 (20.0)	77 (21.6)	1.032 (0.6764, 1.573)		
	Recessive	G/G - G/C	264 (97.8)	353 (98.9)	1	0.4875	0.8126
		C/C	6 (2.2)	4 (1.1)	0.6042 (0.1457, 2.506)		
	Log-additive	—	—	—	0.9887 (0.6769, 1.444)	0.9532	0.995
rs1056827	Dominant	C/C	171 (63.3)	228 (63.9)	1	0.7812	0.9835
		C/A- A/A	99 (36.7)	129 (36.1)	1.052 (0.7341, 1.508)		
	Recessive	C/C - C/A	260 (96.3)	343 (96.1)	1	0.3418	0.8126
		A/A	10 (3.7)	14 (3.9)	1.554 (0.6262, 3.856)		
	Log-additive	—	—	—	1.092 (0.8036, 1.485)	0.5729	0.8718
rs10012	Dominant	G/G	171 (63.3)	230 (64.4)	1	0.9318	0.9835
		G/C-C/C	99 (36.7)	127 (35.6)	1.016 (0.708, 1.458)		
	Recessive	G/G-G/C	260 (96.3)	342 (95.8)	1	0.2845	0.8126
		C/C	10 (3.7)	15 (4.2)	1.631 (0.6658, 3.995)		
	Log-additive	—	—	—	1.072 (0.7899, 1.456)	0.6543	0.8724
rs3813867	Dominant	G/G	171 (63.3)	213 (59.7)	1	0.3651	0.6637
		G/C- C/C	99 (36.7)	144 (40.3)	1.178 (0.8266, 1.678)		
	Recessive	G/G- G/C	258 (95.6)	344 (96.4)	1	0.4491	0.8126
		C/C	12 (4.4)	13 (3.6)	0.7175 (0.3037, 1.695)		
	Log-additive	—	—	—	1.082 (0.7998, 1.463)	0.6103	0.8718
rs2031920	Dominant	C/C	171 (63.3)	213 (59.7)	1	0.3651	0.6637
		C/T-T/T	99 (36.7)	144 (40.3)	1.178 (0.8266, 1.678)		
	Recessive	C/C-C/T	258 (95.6)	344 (96.4)	1	0.4491	0.8126
		T/T	12 (4.4)	13 (3.6)	0.7175 (0.3037, 1.695)		
	Log-additive	—	—	—	1.082 (0.7998, 1.463)	0.6103	0.8718
rs915908	Dominant	G/G	190 (70.4)	252 (70.6)	1	0.7128	0.9835
		G/A-A/A	80 (29.6)	105 (29.4)	1.074 (0.7346, 1.57)		
	Recessive	G/G-G/A	258 (95.6)	345 (96.6)	1	0.8063	0.8959
		A/A	12 (4.4)	12 (3.4)	0.8921 (0.3581, 2.222)		
	Log-additive	—	—	—	1.037 (0.7549, 1.424)	0.8237	0.9691
rs6413432	Dominant	T/T	145 (53.7)	198 (55.5)	1	0.5408	0.9014
		T/A-A/A	125 (46.3)	159 (44.5)	0.8977 (0.6352, 1.269)		
	Recessive	T/T-T/A	248 (91.9)	338 (94.7)	1	0.1336	0.6682
		A/A	22 (8.1)	19 (5.3)	0.5923 (0.2988, 1.174)		
	Log-additive	—	—	—	0.8556 (0.6482, 1.129)	0.2707	0.545

aOR: adjusted odds ration, adjusted for maternal age, gestational week, housing renovation, factory or landfill nearby, cooking at home, parental smoking or ETS exposure, maternal alcohol consumption, folic acid supplements.

Table 4 displays the association between maternal haplotypes and the risk of CHDs. In the AHR gene, the haplotype C-G-A-C was associated with a decreased risk of CHDs (aOR = 0.765, *P* = 0.0486), and the haplotype T-A-G-A was associated with an increased risk of CHDs (aOR = 1.33, *P* = 0.0447). In the CYP1A2 gene, one haplotype block defined by 2 SNPs (rs762551 and rs4646425) showed a significant association with the risk of CHDs (the haplotype A-T: aOR = 1.75, *P* = 0.0239; the haplotype C-C: aOR = 0.706, *P* = 0.00877). No significant association was found between any of other haplotypes and the risk of CHDs. Linkage disequilibrium analysis is shown in Supplementary Appendix C, Figure S1.

**Table 4:** Association between maternal haplotypes and the risk of CHDs

Gene	Haplotype	Frequency			*P*-value	aOR
		All	Controls	Cases		
AHR						
Block1	4-SNPs: rs2158041- rs7811989 - rs2066853- rs2040623					
	**C-G-A-C**	**0.333**	**0.3556**	**0.3137**	**0.0486**	0.765*
	C-G-G-C	0.0434	0.04019	0.04622	0.866	0.948
	**T-A-G-A**	**0.241**	**0.2124**	**0.2633**	**0.0447**	1.33*
	C-G-G-A	0.375	0.3783	0.3739	0.655	1.06
CYP1A1						
Block1	3-SNPs: rs1048943- rs4646422 – rs4646421					
	C-C-A	0.259	0.2548	0.2627	0.695	1.06
	T-C-A	0.177	0.173	0.1799	0.704	1.07
	T-T-G	0.12	0.1302	0.1106	0.273	0.811
	T-C-G	0.44	0.4375	0.4434	0.91	1.01
CYP1A2						
Block1	2-SNPs: rs762551- rs4646425					
	**A-T**	**0.0805**	**0.05926**	**0.09664**	**0.0239**	1.75*
	**C-C**	**0.307**	**0.3444**	**0.2787**	**0.00877**	0.706*
	A-C	0.612	0.5963	0.6246	0.182	1.19
Block2	2-SNPs: rs2472304- rs2470890					
	A-T	0.164	0.1593	0.1681	0.24	1.23
	G-C	0.834	0.8407	0.8291	0.186	0.793
CYP1B1						
Block1	3-SNPs: rs2855658- rs1056837- rs1056836					
	T-A-C	0.107	0.1056	0.1078	0.923	1.02
	C-G-G	0.888	0.8889	0.8866	0.953	1.01
Block2	2-SNPs: rs1056827- rs10012					
	A-C	0.199	0.2019	0.1961	0.732	1.06
	C-G	0.797	0.7981	0.7969	0.504	0.901
CYP2E1						
Block1	3-SNPs: rs3813867- rs2031920- rs915908					
	G-C-A	0.165	0.1704	0.1603	0.864	1.03
	C-T-G	0.212	0.2056	0.2163	0.643	1.07
	G-C-G	0.621	0.6241	0.6199	0.574	0.93

aOR: adjusted odds ration, adjusted for maternal age, gestational week, housing renovation, factory or landfill nearby, cooking at home, parental smoking or ETS exposure, maternal alcohol consumption, folic acid supplements.

### Interaction between maternal genotypes and exposure to PAHs on the risk of CHDs

Assuming a dominant genetic model (minor allele considered to be the risk allele) and a 1 df association test, Table 5 shows the interaction between maternal genotypes and exposure to PAHs and the risk of CHDs. When exposed to the higher level of PAHs, the risk of CHDs for the children of mothers carrying the C/T or T/T genotypes of SNP rs2158041 in the AHR gene was 1.724 (χ^2^ = 7.209, *P* = 0.007) times greater than the aOR for the children of the mothers carrying the C/C genotype. The risk of CHDs for the children of mothers carrying the G/A or A/A genotypes of SNP rs7811989 was 1.735 (χ^2^ = 7.364, *P* = 0.007) times greater than the aOR of the children of mothers carrying the G/G genotype. Multiplicative-scale interactions between maternal exposure to PAHs and the SNP rs4646425 in the CYP1A2 gene (*P* = 0.03) and the SNP rs915908 in the CYP2E1 gene (*P* = 0.0238) and the risk of CHDs were observed. No multiplicative-scale interactions were observed between maternal exposure to PAHs or the other SNPs and the risk of CHDs.

**Table 5:** Interaction between maternal PAHs exposure and genotypes on the risk of CHDs.

Genotype	PAHs exposure	Count (%)		aOR (95% CI)	Interaction Test	
		Controls	Cases		G^2^	*P*
**rs2158041**					3.044	0.081
C/C	low	33 (12.2)	25 (7.0)	Ref.		
C/C	high	135 (50.0)	169 (47.3)	1.421 (0.762,2.648)		
C/T or T/T	low	29 (10.7)	18 (5.0)	0.739 (0.310,1.762)		
C/T or T/T	high	73 (27)	145 (40.6)	2.449 (1.281, 4.682)		
**rs7811989**					3.605	0.0576
G/G	low	34 (12.6)	26 (7.3)	Ref.		
G/G	high	135 (50.0)	168 (47.1)	1.383 (0.749,2.544)		
G/A or A/A	low	28 (10.4)	17 (4.8)	0.687 (0.286,1.648)		
G/A or A/A	high	73 (27.0)	146 (40.9)	2.399 (1.267,4.544)		
**rs2066853**					0.139	0.7093
G/G	low	23 (8.5)	22 (6.2)	Ref.		
G/G	high	88 (32.6)	141 (39.5)	1.815 (0.871,3.781)		
G/A or A/A	low	39 (14.4)	21 (5.9)	0.652 (0.270,1.574)		
G/A or A/A	high	120 (44.4)	173 (48.5)	1.422 (0.693,2.916)		
**rs2040623**					0.09	0.7642
A/A	low	19 (7.0)	18 (5.0)	Ref.		
A/A	high	77 (28.5)	123 (34.5)	1.830 (0.829,4.042)		
A/C or C/C	low	43 (15.9)	25 (7.0)	0.678 (0.274,1.673)		
A/C or C/C	high	131 (48.5)	191 (53.5)	1.443 (0.671,3.105)		
**rs1048943**					0.002	0.9643
T/T	low	34 (12.6)	26 (7.3)	Ref.		
T/T	high	112 (41.5)	168 (47.1)	2.048 (1.098,3.819)		
T/C or C/C	low	28 (10.4)	17 (4.8)	1.021 (0.427,2.439)		
T/C or C/C	high	96 (35.6)	146 (40.9)	2.045 (1.088, 3.844)		
**rs4646422**					0.605	0.4367
C/C	low	49 (18.1)	36 (10.1)	Ref.		
C/C	high	152 (56.3)	248 (69.5)	1.895 (1.117,3.217)		
C/T or T/T	low	13 (4.8)	7 (2.0)	0.487 (0.159,1.490)		
C/T or T/T	high	56 (20.7)	66 (18.5)	1.431 (0.775,2.644)		
**rs4646421**					0.096	0.7567
G/G	low	18 (6.7)	13 (3.6)	Ref.		
G/G	high	66 (24.4)	95 (26.6)	2.275 (0.963,5.372)		
G/A or A/A	low	44 (16.3)	30 (8.4)	1.267 (0.496,3.238)		
G/A or A/A	high	142 (52.6)	173 (61.3)	2.449 (1.079,5.561)		
**rs762551**					1.232	0.267
A/A	low	26 (9.6)	23 (6.4)	Ref.		
A/A	high	93 (34.4)	164 (45.9)	1.519 (0.765,3.018)		
A/C or C/C	low	36 (13.3)	20 (5.6)	0.431 (0.180,1.028)		
A/C or C/C	high	115 (42.6)	450 (42.0)	1.120 (0.567, 2.212)		
**rs4646425**					4.736	0.03
C/C	low	58 (21.5)	32 (9.0)	Ref.		
C/C	high	180 (66.7)	259 (72.5)	2.50 (1.484,4.213)		
C/T or T/T	low	4 (1.5)	11 (3.1)	5.988 (1.588,22.57)		
C/T or T/T	high	28 (10.4)	55 (15.4)	3.441 (1.728,6.853)		
**rs2472304**					0.564	0.4527
G/G	low	41 (15.2)	31 (8.7)	Ref.		
G/G	high	148 (54.8)	213 (59.7)	1.803 (1.036,3.140)		
G/A or A/A	low	21 (7.8)	12 (3.4)	0.852 (0.321,2.258)		
G/A or A/A	high	60 (22.2)	101 (28.3)	2.301 (1.241,4.264)		
**rs2740890**					0.591	0.442
C/C	low	41 (15.2)	31 (8.7)	Ref.		
C/C	high	148 (54.8)	213 (59.7)	1.79 (1.033,3.131)		
C/T or T/T	low	21 (7.8)	12 (3.4)	0.852 (0.322,2.259)		
C/T or T/T	high	60 (22.2)	101 (28.3)	2.317 (1.250,4.295)		
**rs2855658**					0.001	0.9748
C/C	low	49 (18.1)	33 (9.2)	Ref.		
C/C	high	168 (62.2)	250 (70.0)	2.022 (1.184,3.450)		
C/T or T/T	low	13 (4.8)	10 (2.8)	1.067 (0.382,2.979)		
C/T or T/T	high	40 (14.8)	64 (17.9)	2.210 (1.152, 4.240)		
**rs1056837**					0.051	0.8213
G/G	low	48 (17.8)	33 (9.2)	Ref.		
G/G	high	169 (62.6)	249 (69.7)	1.977 (1.157,3.380)		
G/A or A/A	low	14 (5.2)	10 (2.8)	1.015 (0.367,2.805)		
G/A or A/A	high	39 (14.4)	65 (18.2)	2.286 (1.188,4.399)		
**rs1056836**					0.041	0.8395
G/G	low	48 (17.8)	33 (9.2)	Ref.		
G/G	high	168 (62.2)	247 (69.2)	1.981 (1.158,3.387)		
G/C or C/C	low	14 (5.2)	10 (2.8)	1.015 (0.367,2.805)		
G/C or C/C	high	40 (14.8)	67 (18.8)	2.259 (1.179,4.329)		
**rs1056827**					0.683	0.4086
C/C	low	42 (15.6)	25 (7.0)	Ref.		
C/C	high	129 (47.8)	203 (56.9)	2.359 (1.300,4.281)		
C/A or A/A	low	20 (7.4)	18 (5.0)	1.475 (0.605,3.596)		
C/A or A/A	high	79 (29.3)	111 (31.1)	2.304 (1.227,4.326)		
**rs10012**					0.836	0.3605
G/G	low	42 (15.6)	25 (7.0)	Ref.		
G/G	high	129 (47.8)	205 (57.4)	2.398 (1.321,4.350)		
G/C or C/C	low	20 (7.4)	18 (5.0)	1.475 (0.605,3.596)		
G/C or C/C	high	79 (29.3)	109 (30.5)	2.240 (1.192,4.209)		
**rs3813867**					0.477	0.4898
G/G	low	36 (13.3)	26 (7.3)	Ref.		
G/G	high	135 (50)	187 (52.4)	1.780 (0.968,3.273)		
G/C or C/C	low	26 (9.6)	17 (4.8)	0.915 (0.382,2.195)		
G/C or C/C	high	73 (27.0)	127 (35.6)	2.286 (1.204, 4.342)		
**rs2031920**					0.477	0.4898
C/C	low	36 (13.3)	26 (7.3)	Ref.		
C/C	high	135 (50)	187 (52.4)	1.780 (0.968,3.273)		
C/T or T/T	low	26 (9.6)	17 (4.8)	0.915 (0.382,2.195)		
C/T or T/T	high	73 (27.0)	127 (35.6)	2.286 (1.204, 4.342)		
**rs915908**					5.108	0.0238
G/G	low	41 (15.2)	34 (9.5)	Ref.		
G/G	high	149 (55.2)	218 (61.1)	1.448 (0.832,2.520)		
G/A or A/A	low	21 (7.8)	9 (2.5)	0.343 (0.124,0.953)		
G/A or A/A	high	59 (21.9)	96 (26.9)	1.728 (0.931,3.208)		
**rs6413432**					0.003	0.9563
T/T	low	32 (11.9)	23 (6.4)	Ref.		
T/T	high	113 (41.9)	175 (49.0)	2.003 (1.054,3.806)		
T/A or A/A	low	30 (11.1)	20 (5.6)	0.869 (0.367,2.056)		
T/A or A/A	high	95 (35.2)	139 (38.9)	1.783 (0.928,3.427)		

aOR: adjusted odds ration, adjusted for maternal age, gestational week, housing renovation, factory or landfill nearby, cooking at home, parental smoking or ETS exposure, maternal alcohol consumption, folic acid supplements.

## Discussion

In this case-control study, we evaluated the association between maternal exposure to PAHs and the risk of CHDs and the association between maternal genetic variants and the risk of CHDs. We further explored possible interactions between the risk of CHDs and maternal exposure to PAHs and genetic variants.

PAHs are lipophilic, which means that they can pass through the placenta. The estimated transplacental dose of PAHs is about ten times lower than the dose in maternal tissues^12,28^. The developing embryos may be as much as 10 times more susceptible than the mother to PAH-induced DNA damage^12^. Suggested mechanisms of the teratogenicity of PAHs include oxidative stress^29^; changes in signal transduction pathways^30,31^; the formation of bulky PAH-DNA adducts that result in a spectrum of cellular mutations that may be teratogenic^12,32^; or epigenetic changes, including DNA methylation^33^.

Previous studies in experimental model systems have suggested that prenatal exposure to PAHs is associated with CHDs^19,20^. PAHs are important components of PM2.5 and PM10^34^. One human study found that PM10 exposure during weeks 3–8 of pregnancy is associated with isolated atrial septal defects (OR = 2.27, 95% CI: 1.43, 3.60)^35^. Another study suggested that PM10 is associated with increased odds of pulmonary valve stenosis (aOR_Fourth Quartile_ = 2.6, 95% CI: 1.2, 5.7) and perimembranous ventricular septal defects (aOR_Third Quartile_ = 2.1, 95% CI: 1.1, 3.9)^36^. Consistent with these results, our study found that higher maternal level of exposure to PAHs during pregnancy is associated with an increased risk of CHDs or CHDs phenotypic subtypes after adjusting for a series of potential confounders. However, one published US study did not support the association between potential maternal occupational exposure to PAHs and various CHDs subtypes^21^. Because there have been only a limited number of human studies regarding the relationship between maternal exposure to PAHs and CHDs, and the results were inconsistent, more research is necessary to elucidate the potential relations between maternal exposure to PAHs and CHDs.

Genetic polymorphisms in AhR lead to substantial differences in sensitivity to the biochemical and toxic effects of chemical compounds in laboratory animals^37^. There have been a few reports on SNPs in the maternal AHR gene, environmental exposure to PAHs during pregnancy and the impacts on the foetus, but the results have been inconsistent. Two previous studies showed that infants born to continuously smoking mothers with the AhR rs2066853 wild-type genotype had significantly lower estimated birth weights and lengths compared with the offspring of non-smokers^38,39^. One previous study did not observe an association between SNPs (including rs2158041, rs7811989, and rs2040623) of the maternal AHR gene and NTD or between NTD and the interaction between maternal genes and indoor air pollution^26^. Another later study did not find that the AhR rs2066853 polymorphism modified the effect of prenatal dioxin levels on birth size^40^. The polymorphism of the AHR gene affected the expression of the protein^41^. Our study showed that SNPs (including rs2158041, rs7811989, rs2066853, and rs2040623) in the maternal AHR gene were associated with CHDs or CHDs subtypes, but they were not observed after the FDR correction. The haplotypes of the AHR gene were associated with the risk of CHDs. The SNPs rs2158041 and rs7811989 may modify the association of maternal exposure to PAHs with foetal CHDs. The molecular mechanism remains unclear. Perhaps these two intronic polymorphisms influence the alternative spicing of the gene products^42^, or they might be in LD with other causal loci or genes, further affecting the metabolism of PAHs.

Four members of the CYP family, CYP1A1, CYP1A2, CYP1B1 and CYP2E1, are the main Phase I enzymes mediating the toxicity of xenobiotic chemicals, including PAHs. Their polymorphisms are associated with increased risk of many types of cancer, such as renal cell carcinoma, lung cancer, colorectal cancer, and breast cancer^43–46^. Several animal studies have demonstrated that knocking out CYP1A1, CYP1A2, or CYP1B1 expression had no deleterious effects when the mice were raised in a clean environment; however, severe adverse effects appeared in the knockout mice when they were challenged with toxins and carcinogens^47,48^. In human studies, the results regarding the association between maternal gene polymorphisms or gene-environmental interactions and the offspring’s health effects have been inconsistent. One previous study did not find an association between the maternal CYP1A1 rs1048943 polymorphism and the risk of non-syndromic oral cleft^49^. Another study did not find an association between the maternal CYP1B1 rs1056836 polymorphism and childhood medulloblastoma^50^. One study in 2006 showed that the maternal CYP1B1 rs1048943 polymorphism was associated with first-trimester miscarriage and that it might also modify the risk of first-trimester miscarriage among coffee drinkers^51^. One later study in 2013 did not find an association between the maternal CYP1A1 rs1048943 polymorphism and the risk of preterm delivery, but it showed an interaction between a high organochlorine pesticide level and the CYP1A1 rs1048943 polymorphism that might magnify the risk of PTD^52^. One report did not find an association between maternal CYP1A1, CYP1A2, CYP1B1 polymorphisms and neural tube defects, but it found that the maternal CYP1B1 rs2855658 polymorphism modified the effect of indoor air pollution on the risk of neural tube defects^26^. Another study did not find an association between the maternal CYP1A2 rs762551 polymorphism and the risk of gastroschisis or an association between the risk of gastroschisis and gene-maternal smoking interaction^53^. Our study observed that the maternal CYP1A2 rs762551 and rs4646425 polymorphisms are associated with CHDs or CHDs subtypes but that those associations were not observed after the FDR correction. The haplotypes of the CYP1A2 gene were associated with the risk of CHDs. Multiplicative-scale interactions were observed between maternal exposure to PAHs, the SNP rs4646425 in CYP1A2 and the SNP rs915908 in CYP2E, and the risk of CHDs. The molecular mechanism underlying these interactions remains to be determined.

This study has several strengths. First, to our knowledge, this is the first study to evaluate the effect of the interaction between maternal exposure to PAHs during pregnancy and maternal genotypes on the risk of CHDs. Second, we used a urinary biomarker-based approach to examine the relationship between maternal exposure to PAHs and the risk of CHDs, offering an objective measure of exposure to PAHs, in contrast to the previous exposure assessment that relied solely on expert industrial hygienist consensus or the self-report of pregnant women, whose knowledge of their exposure to PAHs is likely to be limited^54^. Finally, the subjects of our study were pregnant women non-occupationally exposed to PAHs; thus, the results can be generalized to all women because the environmental factors can be assessed at the individual level.

Our study is subject to certain limitations. First, given the relatively moderate number of subjects, future studies with larger sample sizes are warranted to confirm or refute our findings. Second, only maternal exposure to PAHs and genetic susceptibilities were considered; however, the genetics of cardiac development is likely to be a complex interplay between both maternal and foetal genetic susceptibilities. Therefore, future studies are needed to investigate the effects of foetal exposure, foetal genotypes, and the interaction between them on the risk of CHDs.

In summary, our findings indicate that higher maternal levels of exposure to PAHs during pregnancy might be associated with increased risk of foetal CHDs and CHDs subtypes. The haplotypes of the AHR or CYP1A2 genes were associated with the risk of CHDs, and the AHR gene rs2158041 and rs7811989 polymorphisms may modify the association of maternal exposure to PAHs with foetal CHDs. The maternal SNPs rs4646425 in CYP1A2 and rs915908 in CYP2E1 significantly interacted with the effect of maternal exposure to PAHs on CHDs.

## Materials and Methods

### Study population

This case–control study was performed from February 2010 to July 2015. The study subjects were recruited from six tertiary maternal and child health hospitals with expertise in foetal echocardiography to screen for foetal CHDs.

The main inclusion criteria were as follows: pregnant women with foetuses diagnosed with CHDs and without any extracardiac abnormalities determined by echocardiography and with gestational age more than 12 weeks. CHDs was confirmed in the foetuses by appointed senior ultrasonic doctors. For the CHDs-affected foetuses that were aborted, CHDs was further confirmed by humanitarian examination of the pathological anatomy. For the CHDs-affected foetuses that were born, a further ultrasound examination was performed within 30 postnatal days. Furthermore, a telephone follow-up was performed within 60 days. The exclusion criteria were as follows: (1) foetuses with syndromic diseases and chromosomal aberrations and (2) woman with multiple pregnancies.

The controls were pregnant women with foetuses with no major congenital malformations diagnosed by echocardiography in the same hospital and gestational age more than 12 weeks. A further ultrasound examination was performed within 30 postnatal days, and a telephone follow-up was performed within 60 days.

Based on the anatomic lesions, CHDs cases were categorized into six subgroups: (1) septal defects, (2) conotruncal heart defects, (3) right-sided obstructive malformations, (4) left-sided obstructive malformations, (5) anomalous pulmonary venous return, and (6) other heart abnormalities^55,56^.

**Figure 1:**
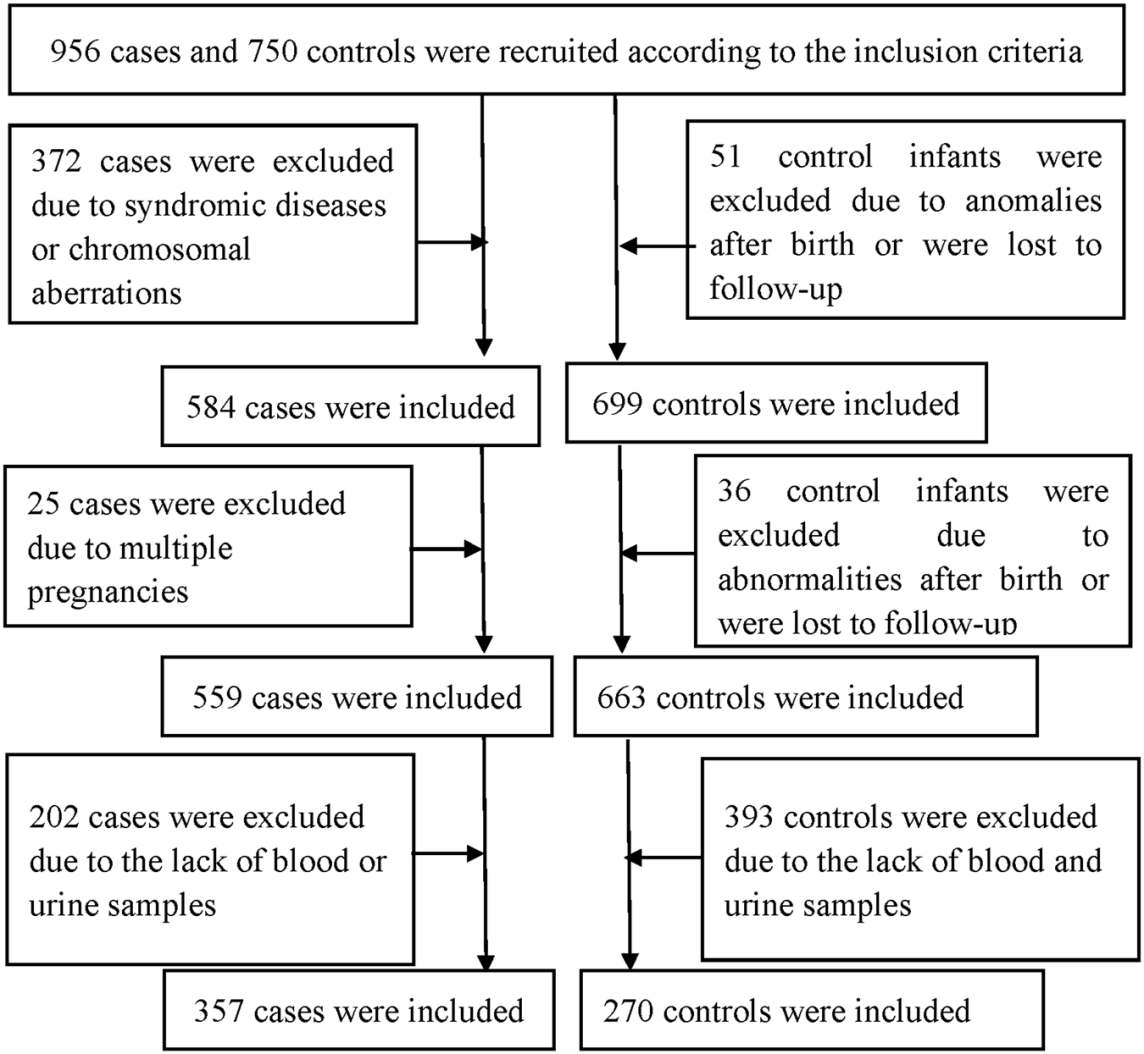
Flowchart presenting the inclusion and exclusion of case and control participants.

During the study period, 956 cases and 750 controls were initially recruited. According to the exclusion criteria, 397 cases and 87 controls were excluded from the study. Additionally, 202 cases and 393 controls were excluded due to a lack of maternal blood or urine samples. Therefore, 357 cases and 270 controls were included. The flowchart of case and control inclusion and exclusion is shown in Fig. 1.

All the participants signed an informed consent form. This research was approved by the Ethics Committee of Sichuan University (No. 2010004) and followed the tenets of the Declaration of Helsinki.

### Data and biological sample collection

Each participant participated in a face-to-face interview when they were recruited during the antenatal examination. The questionnaire comprised eight parts, including parental social demographics, living environment, living habits, working environment, maternal reproductive history, maternal illness and drug use history, maternal diet and nutrition, and maternal life events and mental state. Information on potential confounders was obtained for inclusion as covariate factors.

Referring to the literature, potential confounders are those factors that correlate with both the main determinant and CHDs. These potential confounders included maternal age (at the time of the last menstrual period), gestational week, home or workplace renovation (yes or no), exposure to a factory or landfill nearby (<1000 metres, yes or no), cooking at home (often: ≥4 times/week, never, or occasional: 1~4 times/week), parental smoking or environmental tobacco smoke (ETS) exposure (yes or no), maternal alcohol consumption (often: ≥1 time(s)/week, occasional: <1 time/week, or never), and use of folic acid supplements (yes or no). The age and the gestational week were used as continuous variables in the multivariate analysis.

Ten millilitres of urine was collected from each participant in the morning and stored at −70°C until analysis. Four millilitres of blood was collected in EDTA from each participant by venepuncture and stored at −70°C until genotyping.

### Assessment of exposure to PAHs

1-OHPG concentration was measured in 1 ml urine specimens at the West China School of Public Health, Sichuan University, using ultra-high-performance liquid chromatography coupled with tandem mass spectrometry (UPLC-MS/MS).

The analysis was performed on a Nexera X2 LC-30AD Liquid Chromatograph System (Shimadzu, Japan) coupled with a TSQ Vantage tandem mass spectrometer (Thermo Fisher Scientific, USA) with an electrospray ionization (ESI) interface operated in a negative ion mode. The limit of detection was 0.015 ng 1-OHPG/ml urine. The details regarding the preparation and analysis of the samples are available in Supplementary appendix D.

Some environmental factors are risk factors for disease, but the association is not simply linear^57–59^. In previous studies that conducted an association analysis between environmental exposures and birth defects, researchers generally did not directly incorporate the concentrations of environmental exposures into the models but, rather, grouped environmental exposures according to certain criteria (such as the general population reference value or the normal range) to obtain the odds ratios under different environmental exposures^13,60,61^. In this study, the subjects were non-occupationally exposed pregnant women, who are representative of the general population. We searched many studies but were unable to obtain a reference value for exposure to polycyclic aromatic hydrocarbons or the dose-effect threshold related to CHDs in the general population. Therefore, the Youden Index (sensitivity + specificity − 1) within the receiver operating characteristic (ROC) curve was used to identify the optimal cut-off point used to define the binary exposure to PAHs. The receiver operating characteristic curve and area are shown in Supplementary Appendix E, Figure S2 and Table S5. When the Youden Index reached the maximum value of 0.10918, the concentration of 1-OHPG was 0.03186 µg/g Cr. Therefore, maternal exposure to PAHs was categorized into two groups: “high” if the 1-OHPG concentration was above 0.03186 µg/g Cr and “low” if the 1-OHPG concentration was less than or equal to 0.03186 µg/g Cr.

### DNA extraction and genotyping

Genomic DNA was extracted from peripheral blood leukocytes using a QIAamp DNA Blood Mini Kit (Qiagen, Cat. No. 51106, Germany) according to the recommended protocol.

SNPs in the AHR, CYP1A1, CYP1A2, CYP1B1, and CYP2E1 genes were selected based on the following principal criteria: (1) an association with diseases in previous studies or the metabolic level of PAHs^26,43–46,62–70^ and (2) a minor allele frequency >0.05 in Han Chinese. In total, 20 SNPs were selected. The SNPs were genotyped using an improved multiplex ligation detection reaction (iMLDR) technique that was newly developed by Genesky Biotechnologies Inc. (Shanghai, China). More detailed information about the studied genetic variants and genotyping is presented in Supplementary Appendix F, Table S5.

For quality-control assessment, genotyping was repeated in 10% of samples, and the consistency rate was 100%.

### Statistical Analysis

Chi-square statistics tested the differences in covariates between the cases and controls. Unconditional logistic regression analysis was performed to investigate the association between maternal exposure to PAHs and foetal CHDs using Statistical Package for Social Sciences (SPSS) version 16.0 software (SPSS Inc., IBM, Chicago, USA).

Hardy–Weinberg equilibrium was assessed in the controls using Plink software (http://pngu.mgh.harvard.edu/~purcell/plink/). The pairwise linkage disequilibrium (LD) patterns and haplotype structures of CYP1A1, CYP1A2, CYP1B1, CYP2E1 and AHR genes were analysed using Haploview 4.2 software. Unconditional logistic regression analysis was performed to investigate the association between individual genetic polymorphisms and CHDs using Plink software.

The effects of the gene-exposure interactions on CHDs occurrence were evaluated by logistic models using SPSS version 16.0 software (SPSS Inc., IBM, Chicago, USA).

All analyses were adjusted for covariates/potential confounders. False discovery rate (FDR) correction of multiple hypothesis testing was performed. Two-sided *P* < 0.05 was considered statistically significant.

## Electronic supplementary material

Supplementary file
