# Supplementary material for: Assessment of interaction between maternal polycyclic aromatic hydrocarbons exposure and genetic polymorphisms on the risk of congenital heart diseases
Source: Sci Rep. 2018 Feb 15;8:3075. doi: 10.1038/s41598-018-21380-3 (PMC5814464; doi:10.1038/s41598-018-21380-3)
Supplement: Supplementary file 1 — Supplementary file [file 41598_2018_21380_MOESM1_ESM.doc]

**Assessment of interaction between maternal polycyclic aromatic hydrocarbons**

**exposure and genetic polymorphisms on the risk of congenital heart diseases**

Nana Li ^1,2,*^, Yi Mu ^1,2,*^,Zhen Liu^1,2^,, Ying Deng^1,2^,, Yixiong Guo^1,2^,, Xuejuan Zhang^3^, Xiaohong Li ^1,2^,, Ping Yu^1,2^,, Yanping Wang^1,2^, &Jun Zhu^1,2^

**Appendix A:**

**Table S1** Hardy-Weinberg equilibrium evaluation of SNPs

| Gene | dbSNP_ID | Group | Genotype count（frequency %） | | | HWE *P* |
| --- | --- | --- | --- | --- | --- | --- |
| AHR | rs2158041 |  | C/C | C/T | T/T |  |
|  |  | Controls | 168（62.2） | 85（31.5） | 17（6.2） | 0.1601 |
|  |  | Cases | 194（54.3） | 137（38.4） | 26（7.3） |  |
|  | rs7811989 |  | G/G | G/A | A/A |  |
|  |  | Controls | 169（62.6） | 83（30.7） | 18（6.7） | 0.1087 |
|  |  | Cases | 194（54.3） | 137（38.4） | 26（7.3） |  |
|  | rs2066853 |  | G/G | G/A | A/A |  |
|  |  | Controls | 111（41.1） | 126（46.7） | 33（12.2） | 0.8943 |
|  |  | Cases | 163（44.9） | 163（45.5） | 31（9.6） |  |
|  | rs2040623 |  | A/A | A/C | C/C |  |
|  |  | Controls | 96（35.6） | 132（48.9） | 42（15.6） | 0.8009 |
|  |  | Cases | 141（39.5） | 173（48.5） | 43（12.0） |  |
| CYP1A1 | rs1048943 |  | T/T | T/C | C/C |  |
|  |  | Controls | 146（54.1） | 108（40.0） | 16（5.9） | 0.634 |
|  |  | Cases | 194（54.3） | 136（38.1） | 27（7.6） |  |
|  | rs4646422 |  | C/C | C/T | T/T |  |
|  |  | Controls | 201（74.4） | 67（24.8） | 2（0.7） | 0.2799 |
|  |  | Cases | 284（79.6） | 67（18.8） | 6（1.7） |  |
|  | rs4642421 |  | G/G | G/A | A/A |  |
|  |  | Controls | 84（31.1） | 141（52.2） | 45（16.7） | 0.3202 |
|  |  | Cases | 108（30.3） | 182（51.0） | 67（18.8） |  |
| CYP1A2 | rs762551 |  | A/A | A/C | C/C |  |
|  |  | Controls | 119（44.1） | 116（43.0） | 35（13.0） | 0.4207 |
|  |  | Cases | 187（52.4） | 141（39.5） | 29（8.1） |  |
|  | rs4646425 |  | C/C | C/T | T/T |  |
|  |  | Controls | 238（88.1） | 32（11.9） | 0（0.0） | 0.609 |
|  |  | Cases | 291（81.5） | 63（17.6） | 3（0.8） |  |
|  | rs2472304 |  | G/G | G/A | A/A |  |
|  |  | Controls | 189（70.0） | 76（28.1） | 5（1.9） | 0.5003 |
|  |  | Cases | 244（68.3） | 105（29.4） | 8（2.2） |  |
|  | rs2470890 |  | C/C | C/T | T/T |  |
|  |  | Controls | 189（70.0） | 76（28.1） | 5（1.9） | 0.5003 |
|  |  | Cases | 244（68.3） | 105（29.4） | 8（2.2） |  |
| CYP1B1 | rs2855658 |  | C/C | C/T | T/T |  |
|  |  | Controls | 217（80.4） | 48（17.8） | 5（1.9） | 0.2082 |
|  |  | Cases | 283（79.3） | 70（19.6） | 4（1.1） |  |
|  | rs1056837 |  | G/G | G/A | A/A |  |
|  |  | Controls | 217（80.4） | 47（17.4） | 6（2.2） | 0.1069 |
|  |  | Cases | 282（79.0） | 71（19.9） | 4（1.1） |  |
|  | rs1056836 |  | G/G | G/C | C/C |  |
|  |  | Controls | 216（80.0） | 48（17.8） | 6（2.2） | 0.1154 |
|  |  | Cases | 280（78.4） | 73（20.4） | 4（1.1） |  |
|  | rs1056827 |  | C/C | C/A | A/A |  |
|  |  | Controls | 171（63.3） | 89（33.0） | 10（3.7） | 0.8505 |
|  |  | Cases | 228（63.9） | 115（32.2） | 14（3.9） |  |
|  | rs10012 |  | G/G | G/C | C/C |  |
|  |  | Controls | 171（63.3） | 89（33.0） | 10（3.7） | 0.8505 |
|  |  | Cases | 230（64.4） | 112（31.4） | 15（4.2） |  |
| CYP2E1 | rs3813867 |  | G/G | G/C | C/C |  |
|  |  | Controls | 171（63.3） | 87（32.2） | 12（4.4） | 0.852 |
|  |  | Cases | 213（59.7） | 131（36.7） | 13（3.6） |  |
|  | rs2031920 |  | C/C | C/T | T/T |  |
|  |  | Controls | 171（63.3） | 87（32.2） | 12（4.4） | 0.852 |
|  |  | Cases | 213（59.7） | 131（36.7） | 13（3.6） |  |
|  | rs915908 |  | G/G | G/A | A/A |  |
|  |  | Controls | 190（70.4） | 68（25.2） | 12（4.4） | 0.08305 |
|  |  | Cases | 252（70.6） | 93（26.1） | 12（3.4） |  |
|  | rs6413432 |  | T/T | T/A | A/A |  |
|  |  | Controls | 145（53.7） | 103（38.1） | 22（8.1） | 0.5402 |
|  |  | Cases | 198（55.5） | 140（39.2） | 19（5.3） |  |

**Appendix B:**

**Table S2-1 Association between maternal genotypes and the risk of septal defects**

| dbSNP_ID | Model | Genotype | Controls | **septal defects** | aOR(95%CI) | *P*-value | FDR-BH *P* value |
| --- | --- | --- | --- | --- | --- | --- | --- |
|  |  |  | N(%) | N(%) |  |  |  |
| rs2158041 | Dominant | C/C | 168（62.2） | 120（51.1） | 1 | 0.009341 | 0.05361 |
|  |  | C/T- T/T | 102（37.8） | 115（48.9） | **1.674 ( 1.135, 2.469)*** |  |  |
|  | Recessive | C/C- C/T | 253（93.7） | 215（91.5） | 1 | 0.331 | 0.8702 |
|  |  | T/T | 17（6.3） | 20（8.5） | 1.429 (0.6959, 2.933) |  |  |
|  | Log-additive | - | - | - | **1.46 ( 1.076, 1.981 )*** | 0.015 | 0.05806 |
| rs7811989 | Dominant | G/G | 169（62.6） | 120（51.1） | 1 | 0.008305 | 0.05361 |
|  |  | G/A-A/A | 101（37.4） | 115（48.9） | **1.69 ( 1.145, 2.495)*** |  |  |
|  | Recessive | G/G-G/A | 252（93.3） | 215（91.5） | 1 | 0.4287 | 0.8702 |
|  |  | A/A | 18（6.7） | 20（8.5） | 1.331 (0.6555, 2.703) |  |  |
|  | Log-additive | - | - | - | **1.445 ( 1.067, 1.957 )*** | 0.01742 | 0.05806 |
| rs2066853 | Dominant | G/G | 111（41.1） | 115（48.9） | 1 | 0.0352 | 0.1173 |
|  |  | G/A-A/A | 159（58.9） | 120（51.1） | **0.6588 (0.4467,0.9715)*** |  |  |
|  | Recessive | G/G-G/A | 237（87.8） | 216（91.9） | 1 | 0.0632 | 0.8702 |
|  |  | A/A | 33（12.2） | 19（8.1） | 0.5421 (0.2841,1.034) |  |  |
|  | Log-additive | - | - | - | **0.6893 (0.5122,0.9276)*** | **0.01405** | **0.05806** |
| rs2040623 | Dominant | A/A | 96（35.6） | 101（43.0） | 1 | 0.02413 | 0.09653 |
|  |  | A/C-C/C | 174（64.4） | 134（57.0） | **0.6323 (0.4245, 0.9418)*** |  |  |
|  | Recessive | A/A-A/C | 228（84.4） | 207（88.1） | 1 | 0.126 | 0.8702 |
|  |  | CC | 42（15.6） | 28（11.9） | 0.6459(0.369, 1.131) |  |  |
|  | Log-additive | - | - | - | **0.7055 (0.5298, 0.9394)*** | **0.01697** | **0.05806** |
| rs1048943 | Dominant | T/T | 146（54.1） | 130（55.3） | 1 | 0.8171 | 0.9607 |
|  |  | T/C- C/C | 124（45.9） | 105（44.7） | 0.9559 (0.6521, 1.401) |  |  |
|  | Recessive | T/T- T/C | 254（94.1） | 218（92.8） | 1 | 0.4647 | 0.8702 |
|  |  | C/C | 16（5.9） | 17（7.2） | 1.337 (0.6138, 2.912) |  |  |
|  | Log-additive | - | - | - | 1.017 (0.7452, 1.387) | 0.9172 | 0.9655 |
| rs4646422 | Dominant | C/C | 201（74.4） | 190（80.9） | 1 | 0.05577 | 0.1593 |
|  |  | C/T-T/T | 69（25.6） | 45（19.1） | 0.6362(0.4002,1.011) |  |  |
|  | Recessive | C/C-C/T | 268（99.3） | 231（98.3） | 1 | 0.625 | 0.8702 |
|  |  | T/T | 2（0.7） | 4（1.7） | 1.556(0.2643,9.162) |  |  |
|  | Log-additive | - | - | - | 0.7025(0.4601,1.072) | 0.1018 | 0.2909 |
| rs4642421 | Dominant | G/G | 84（31.1） | 75（31.9） | 1 | 0.8356 | 0.9607 |
|  |  | G/A-A/A | 186（68.9） | 160（68.1） | 0.9573(0.6341,1.445) |  |  |
|  | Recessive | G/G-G/A | 225（83.3） | 195（83.0） | 1 | 0.7396 | 0.8702 |
|  |  | A/A | 45（16.7） | 40（17.0） | 1.09(0.6547,1.816) |  |  |
|  | Log-additive | - | - | - | 1.006 (0.759,1.333) | 0.9669 | 0.9669 |
| rs762551 | Dominant | A/A | 119（44.1） | 128（54.5） | 1 | 0.007974 | 0.05361 |
|  |  | A/C- C/C | 151（55.9） | 107（45.5） | **0.5935 (0.4037, 0.8726)*** |  |  |
|  | Recessive | A/A- A/C | 235（87.0） | 213（90.6） | 1 | 0.2332 | 0.8702 |
|  |  | C/C | 35（13.0） | 9.4 | 0.692 (0.3778, 1.268) |  |  |
|  | Log-additive | - | - | - | **0.6951 (0.5225, 0.9247)*** | **0.0125** | **0.05806** |
| rs4646425 | Dominant | C/C | 238（88.1） | 187（79.6） | 1 | 0.01072 | 0.05361 |
|  |  | C/T-T/T | 32（11.9） | 48（20.4） | **1.999 (1.174, 3.404)*** |  |  |
|  | Recessive | C/C-C/T | 270（100.0） | 233（99.1） | 1 | 0.999 | 0.999 |
|  |  | T/T | 0（0.0） | 2（0.9） | 1.078e+009(0,inf) |  |  |
|  | Log-additive | - | - | - | **2.021(1.2,3.403)*** | **0.008167** | **0.05806** |
| rs2472304 | Dominant | G/G | 189（70.0） | 160（68.1） | 1 | 0.1812 | 0.4531 |
|  |  | G/A-A/A | 81（30.0） | 75（31.9） | 1.331(0.8753,2.024) |  |  |
|  | Recessive | G/G-G/A | 265（98.1） | 229（97.4） | 1 | 0.7059 | 0.8702 |
|  |  | A/A | 5（1.9） | 6（2.6） | 1.285(0.3492,4.731) |  |  |
|  | Log-additive | - | - |  | 1.285(0.8821,1.873) | 0.1913 | 0.4781 |
| rs2470890 | Dominant | G/G | 189（70.0） | 161（68.5） | 1 | 0.2139 | 0.4754 |
|  |  | G/A-A/A | 81（30.0） | 74（31.5） | 1.305(0.8576,1.986) |  |  |
|  | Recessive | G/G-G/A | 265（98.1） | 229（97.4） | 1 | 0.7059 | 0.8702 |
|  | Log-additive | A/A  - | 5（1.9）  - | 6（2.6）  - | 1.285(0.3492,4.731)  1.265 (0.8677,1.844) |  |  |
|  |  |  |  |  |  | 0.2218 | 0.4928 |
| rs2855658 | Dominant | C/C | 217（80.4） | 191（81.3） | 1 | 0.6517 | 0.931 |
|  |  | C/T- T/T | 53（19.6） | 44（18.7） | 0.8952 (0.5536, 1.448) |  |  |
|  | Recessive | C/C - C/T | 265（98.1） | 232（98.7） | 1 | 0.9345 | 0.9837 |
|  |  | T/T | 5（1.9） | 3（1.3） | 1.068 (0.221, 5.162) |  |  |
|  | Log-additive | - | - | - | 0.9193 (0.597, 1.416) | 0.7023 | 0.8779 |
| rs1056837 | Dominant | G/G | 217（80.4） | 191（81.3） | 1 | 0.7307 | 0.9607 |
|  |  | G/A-A/A | 53（19.6） | 44（18.7） | 0.919 (0.5683, 1.486) |  |  |
|  | Recessive | G/G-G/A | 265（98.1） | 232（98.7） | 1 | 0.6018 | 0.8702 |
|  |  | A/A | 6（1.9） | 3（1.3） | 0.6601 (0.1386, 3.143) |  |  |
|  | Log-additive | - | - | - | 0.9059 (0.5908, 1.389) | 0.6505 | 0.8674 |
| rs1056836 | Dominant | G/G | 216（80.0） | 191（81.3） | 1 | 0.6176 | 0.931 |
|  |  | G/C- C/C | 54（20.0） | 44（18.7） | 0.8851 (0.5481, 1.429) |  |  |
|  | Recessive | G/G - G/C | 264（97.8） | 232（98.7） | 1 | 0.6018 | 0.8702 |
|  |  | C/C | 6（2.2） | 3（1.3） | 0.6601 (0.1386, 3.143) |  |  |
|  | Log-additive | - | - | - | 0.8793 (0.5737, 1.348) | 0.5549 | 0.8674 |
| rs1056827 | Dominant | C/C | 171（63.3） | 152（64.7） | 1 | 0.9127 | 0.9607 |
|  |  | C/A- A/A | 99（36.7） | 83（35.3） | 1.023 (0.6855, 1.526) |  |  |
|  | Recessive | C/C - C/A | 260（96.3） | 228（97.0） | 1 | 0.6439 | 0.8702 |
|  |  | A/A | 10（3.7） | 7（3.0） | 1.288 (0.4408, 3.761) |  |  |
|  | Log-additive | - | - | - | 1.044 (0.7369, 1.48) | 0.8074 | 0.9111 |
| rs10012 | Dominant | G/G | 171（63.3） | 153（65.1） | 1 | 0.9912 | 0.9912 |
|  |  | G/C-C/C | 99（36.7） | 82（34.9） | 1.002 (0.671, 1.497) |  |  |
|  | Recessive | G/G-G/C | 260（96.3） | 227（96.6） | 1 | 0.5089 | 0.8702 |
|  |  | C/C | 10（3.7） | 8（3.4） | 1.418(0.5033, 3.993) |  |  |
|  | Log-additive | - | - | - | 1.041(0.7361,1.472) | 0.82 | 0.9111 |
| rs3813867 | Dominant | G/G | 171（63.3） | 137（58.3） | 1 | 0.2907 | 0.5286 |
|  |  | G/C- C/C | 99（36.7） | 98（41.7） | 1.234 (0.8356, 1.822) |  |  |
|  | Recessive | G/G- G/C | 258（95.6） | 228（97.0） | 1 | 0.2761 | 0.8702 |
|  |  | C/C | 12（4.4） | 7（3.0） | 0.5716 (0.2089, 1.564) |  |  |
|  | Log-additive | - | - | - | 1.094 (0.7839, 1.528) | 0.5962 | 0.8674 |
| rs2031920 | Dominant | C/C | 171（63.3） | 137（58.3） | 1 | 0.2907 | 0.5286 |
|  |  | C/T-T/T | 99（36.7） | 98（41.7） | 1.234 (0.8356, 1.822) |  |  |
|  | Recessive | C/C-C/T | 258（95.6） | 228（97.0） | 1 | 0.2761 | 0.8702 |
|  |  | T/T | 12（4.4） | 7（3.0） | 0.5716 (0.2089, 1.564) |  |  |
|  | Log-additive | - | - | - | 1.094 (0.7839, 1.528) | 0.5962 | 0.8674 |
| rs915908 | Dominant | G/G | 190（70.4） | 165（70.2） | 1 | 0.6108 | 0.931 |
|  |  | G/A-A/A | 80（29.6） | 70（29.8） | 1.115(0.7324,1.699) |  |  |
|  | Recessive | G/G-G/A | 258（95.6） | 226（96.2） | 1 | 0.8153 | 0.9059 |
|  |  | A/A | 12（4.4） | 9（3.8） | 1.126(0.417,3.039) |  |  |
|  | Log-additive | - | - | - | 1.095(0.7714,1.553) | 0.6127 | 0.8674 |
| rs6413432 | Dominant | T/T | 145（53.7） | 126（53.6） | 1 | 0.8778 | 0.9607 |
|  |  | T/A-A/A | 125（46.3） | 109（46.4） | 0.9704(0.6618,1.423) |  |  |
|  | Recessive | T/T-T/A | 248（91.9） | 223（94.9） | 1 | 0.1678 | 0.8702 |
|  |  | A/A | 22（8.1） | 12（5.1） | 0.5818(0.2694,1.256) |  |  |
|  | Log-additive | - | - | - | 0.8969 (0.66,1.219) | 0.4868 | 0.8674 |

**Table S2-2 Association between maternal genotypes and the risk of conotruncal heart defects**

| dbSNP_ID | Model | Genotype | Controls | **conotruncal heart defects** | aOR(95%CI) | *P*-value | FDR-BH *P* value |
| --- | --- | --- | --- | --- | --- | --- | --- |
|  |  |  | N(%) | N(%) |  |  |  |
| rs2158041 | Dominant | C/C | 168（62.2） | 83（51.9） | 1 | 0.03391 | 0.2226 |
|  |  | C/T- T/T | 102（37.8） | 77（48.1） | **1.613 (1.037, 2.508) *** |  |  |
|  | Recessive | C/C- C/T | 253（93.7） | 143（89.4） | 1 | 0.07125 | 0.475 |
|  |  | T/T | 17（6.3） | 17（10.6） | 2.01 (0.9414, 4.291) |  |  |
|  | Log-additive | - | - | - | **1.505 (1.076 , 2.103)*** | 0.01679 | 0.126 |
| rs7811989 | Dominant | G/G | 169（62.6） | 82（51.3） | 1 | 0.02988 | 0.2226 |
|  |  | G/A-A/A | 101（37.4） | 78（48.7） | **1.633 (1.049, 2.542) *** |  |  |
|  | Recessive | G/G-G/A | 252（93.3） | 143（89.4） | 1 | 0.1043 | 0.5213 |
|  |  | A/A | 18（6.7） | 17（10.6） | 1.857(0.88, 3.92) |  |  |
|  | Log-additive | - | - | - | **1.49 (1.068 , 2.079)*** | 0.0189 | 0.126 |
| rs2066853 | Dominant | G/G | 111（41.1） | 76（47.5） | 1 | 0.1442 | 0.4806 |
|  |  | G/A-A/A | 159（58.9） | 84（52.5） | 0.7214 (0.4653,1.118) |  |  |
|  | Recessive | G/G-G/A | 237（87.8） | 150（93.8） | 1 | 0.0201 | 0.2736 |
|  |  | A/A | 33（12.2） | 10（6.2） | **0.3838 (0.1712, 0.8606)*** |  |  |
|  | Log-additive | - | - | - | **0.6794 (0.4824, 0.9568)*** | **0.02691** | **0.1309** |
| rs2040623 | Dominant | A/A | 96（35.6） | 65（40.6） | 1 | 0.2029 | 0.5073 |
|  |  | A/C-C/C | 174（64.4） | 95（59.4） | 0.7456 (0.4745, 1.172) |  |  |
|  | Recessive | A/A-A/C | 228（84.4） | 146（91.3） | 1 | 0.02736 | 0.2736 |
|  |  | CC | 42（15.6） | 14（8.7） | **0.456 (0.227, 0.916)*** |  |  |
|  | Log-additive | - | - | - | **0.7037 (0.5038, 0.9829)*** | **0.03927** | **0.1309** |
| rs1048943 | Dominant | T/T | 146（54.1） | 82（51.3） | 1 | 0.6921 | 0.9679 |
|  |  | T/C- C/C | 124（45.9） | 78（48.7） | 1.092 (0.7074, 1.685) |  |  |
|  | Recessive | T/T- T/C | 254（94.1） | 147（91.9） | 1 | 0.6623 | 0.9461 |
|  |  | C/C | 16（5.9） | 13（8.1） | 1.213 (0.5099, 2.886) |  |  |
|  | Log-additive | - | - | - | 1.093(0.7693, 1.554) | 0.6189 | 0.8252 |
| rs4646422 | Dominant | C/C | 201（74.4） | 129（80.6） | 1 | 0.1692 | 0.4835 |
|  |  | C/T-T/T | 69（25.6） | 31（19.4） | 0.6898(0.4062,1.171) |  |  |
|  | Recessive | C/C-C/T | 268（99.3） | 156（97.5） | 1 | 0.4054 | 0.8105 |
|  |  | T/T | 2（0.7） | 4（2.5） | 2.137(0.3572,12.79) |  |  |
|  | Log-additive | - | - | - | 0.7837 (0.4883,1.258) | 0.3127 | 0.6862 |
| rs4642421 | Dominant | G/G | 84（31.1） | 43（26.9） | 1 | 0.4318 | 0.7827 |
|  |  | G/A-A/A | 186（68.9） | 117（73.1） | 1.212 (0.7506,1.957) |  |  |
|  | Recessive | G/G-G/A | 225（83.3） | 126（78.8） | 1 | 0.4065 | 0.8105 |
|  |  | A/A | 45（16.7） | 34（21.2） | 1.267(0.7245,2.216) |  |  |
|  | Log-additive | - | - | - | 1.177 (0.8547,1.622) | 0.3176 | 0.6862 |
| rs762551 | Dominant | A/A | 119（44.1） | 84（52.5） | 1 | 0.04453 | 0.2226 |
|  |  | A/C- C/C | 151（55.9） | 76（47.5） | **0.6404 (0.4146, 0.9892)*** |  |  |
|  | Recessive | A/A- A/C | 235（87.0） | 147（91.9） | 1 | 0.1643 | 0.6572 |
|  |  | C/C | 35（13.0） | 13（8.1） | 0.598 (0.2898, 1.234) |  |  |
|  | Log-additive | - | - | - | **0.6986(0.5026, 0.971)*** | **0.03275** | **0.1309** |
| rs4646425 | Dominant | C/C | 238（88.1） | 127（79.4） | 1 | 0.01521 | 0.2226 |
|  |  | C/T-T/T | 32（11.9） | 33（20.6） | **2.093(1.153,3.799)*** |  |  |
|  | Recessive | C/C-C/T | 270（100.0） | 159（99.4） | 1 | 0.9993 | 0.9993 |
|  |  | T/T | 0（0.0） | 1（0.6） | 3.073e+009(0,inf) |  |  |
|  | Log-additive | - | - | - | **2.126 (1.188,3.805)*** | **0.01108** | **0.126** |
| rs2472304 | Dominant | G/G | 189（70.0） | 119（74.4） | 1 | 0.975 | 0.9904 |
|  |  | G/A-A/A | 81（30.0） | 41（25.6） | 0.9922(0.6086,1.618) |  |  |
|  | Recessive | G/G-G/A | 265（98.1） | 157（98.1） | 1 | 0.7888 | 0.986 |
|  |  | A/A | 5（1.9） | 3（1.9） | 1.236(0.2621,5.829) |  |  |
|  | Log-additive | - | - | - | 1.01 (0.6503,1.57) | 0.9631 | 0.9631 |
| rs2470890 | Dominant | G/G | 189（70.0） | 119（74.4） | 1 | 0.975 | 0.9904 |
|  |  | G/A-A/A | 81（30.0） | 41（25.6） | 0.9922(0.6086,1.618) |  |  |
|  | Recessive | G/G-G/A | 265（98.1） | 157（98.1） | 1 | 0.7888 | 0.986 |
|  | Log-additive | A/A  - | 5（1.9）  - | 3（1.9）  - | 1.236(0.2621,5.829)  1.01 (0.6503,1.57) |  |  |
|  |  |  |  |  |  | 0.9631 | 0.9631 |
| rs2855658 | Dominant | C/C | 217（80.4） | 131（81.9） | 1 | 0.3556 | 0.7827 |
|  |  | C/T- T/T | 53（19.6） | 29（18.1） | 0.771 (0.444, 1.339) |  |  |
|  | Recessive | C/C - C/T | 265（98.1） | 158（98.8） | 1 | 0.9822 | 0.9993 |
|  |  | T/T | 5（1.9） | 2（1.2） | 0.9791 (0.1535, 6.246) |  |  |
|  | Log-additive | - | - | - | 0.8072(0.4893, 1.332) | 0.4016 | 0.6862 |
| rs1056837 | Dominant | G/G | 217（80.4） | 130（81.3） | 1 | 0.4696 | 0.7827 |
|  |  | G/A-A/A | 53（19.6） | 30（18.7） | 0.8167 (0.4717, 1.414) |  |  |
|  | Recessive | G/G-G/A | 265（98.1） | 158（98.8） | 1 | 0.5268 | 0.8105 |
|  |  | A/A | 6（1.9） | 2（1.2） | 0.5559 (0.09021, 3.426) |  |  |
|  | Log-additive | - | - | - | 0.8144(0.4988, 1.33) | 0.4117 | 0.6862 |
| rs1056836 | Dominant | G/G | 216（80.0） | 129（80.6） | 1 | 0.4622 | 0.7827 |
|  |  | G/C- C/C | 54（20.0） | 31（19.4） | 0.8157 (0.474, 1.404) |  |  |
|  | Recessive | G/G - G/C | 264（97.8） | 158（98.8） | 1 | 0.5268 | 0.8105 |
|  |  | C/C | 6（2.2） | 2（1.2） | 0.5559 (0.09021, 3.426) |  |  |
|  | Log-additive | - | - | - | 0.8134(0.5001, 1.323) | 0.4053 | 0.6862 |
| rs1056827 | Dominant | C/C | 171（63.3） | 102（63.8） | 1 | 0.8054 | 0.9904 |
|  |  | C/A- A/A | 99（36.7） | 58（36.2） | 1.058 (0.6753, 1.658) |  |  |
|  | Recessive | C/C - C/A | 260（96.3） | 153（95.6） | 1 | 0.356 | 0.8105 |
|  |  | A/A | 10（3.7） | 7（4.4） | 1.668 (0.5627, 4.946) |  |  |
|  | Log-additive | - | - | - | 1.107(0.7559, 1.622) | 0.6004 | 0.8252 |
| rs10012 | Dominant | G/G | 171（63.3） | 104（65.0） | 1 | 0.9904 | 0.9904 |
|  |  | G/C-C/C | 99（36.7） | 56（35.0） | 0.9972(0.6346,1.567) |  |  |
|  | Recessive | G/G-G/C | 260（96.3） | 153（95.6） | 1 | 0.356 | 0.8105 |
|  |  | C/C | 10（3.7） | 7（4.4） | 1.668 (0.5627, 4.946) |  |  |
|  | Log-additive | - | - | - | 1.061 (0.7232,1.557) | 0.761 | 0.8836 |
| rs3813867 | Dominant | G/G | 171（63.3） | 98（61.3） | 1 | 0.726 | 0.9679 |
|  |  | G/C- C/C | 99（36.7） | 62（38.7） | 1.082 (0.6951, 1.685) |  |  |
|  | Recessive | G/G- G/C | 258（95.6） | 152（95.0） | 1 | 0.9371 | 0.9993 |
|  |  | C/C | 12（4.4） | 8（5.0） | 0.9611 (0.3588, 2.574) |  |  |
|  | Log-additive | - | - | - | 1.05(0.7287, 1.512) | 0.7952 | 0.8836 |
| rs2031920 | Dominant | C/C | 171（63.3） | 98（61.3） | 1 | 0.726 | 0.9679 |
|  |  | C/T-T/T | 99（36.7） | 62（38.7） | 1.082 (0.6951, 1.685) |  |  |
|  | Recessive | C/C-C/T | 258（95.6） | 152（95.0） | 1 | 0.9371 | 0.9993 |
|  |  | T/T | 12（4.4） | 8（5.0） | 0.9611 (0.3588, 2.574) |  |  |
|  | Log-additive | - | - | - | 1.05(0.7287, 1.512) | 0.7952 | 0.8836 |
| rs915908 | Dominant | G/G | 190（70.4） | 105（65.6） | 1 | 0.09463 | 0.3785 |
|  |  | G/A-A/A | 80（29.6） | 55（34.4） | 1.493(0.9331,2.39) |  |  |
|  | Recessive | G/G-G/A | 258（95.6） | 152（95.0） | 1 | 0.5025 | 0.8105 |
|  |  | A/A | 12（4.4） | 8（5.0） | 1.439(0.4964,4.174) |  |  |
|  | Log-additive | - | - | - | 1.377(0.9354,2.028) | 0.1048 | 0.2996 |
| rs6413432 | Dominant | T/T | 145（53.7） | 86（53.8） | 1 | 0.8431 | 0.9904 |
|  |  | T/A-A/A | 125（46.3） | 74（46.2） | 0.9572(0.6204,1.477) |  |  |
|  | Recessive | T/T-T/A | 248（91.9） | 150（93.8） | 1 | 0.3666 | 0.8105 |
|  |  | A/A | 22（8.1） | 10（6.7） | 0.6814(0.2963,1.567) |  |  |
|  | Log-additive | - | - | - | 0.9106 (0.6476,1.28) | 0.5903 | 0.8252 |

**Table S2-3 Association between maternal genotypes and the risk of right-sided obstructive malformations**

| dbSNP_ID | Model | Genotype | Controls | **right-sided obstructive malformations** | aOR(95%CI) | *P*-value | FDR-BH *P* value |
| --- | --- | --- | --- | --- | --- | --- | --- |
|  |  |  | N(%) | N(%) |  |  |  |
| rs2158041 | Dominant | C/C | 168（62.2） | 60（52.6） | 1 | 0.07573 | 0.3856 |
|  |  | C/T- T/T | 102（37.8） | 54（47.4） | 1.545 (0.9559, 2.498) |  |  |
|  | Recessive | C/C- C/T | 253（93.7） | 106（93.0） | 1 | 0.5064 | 0.7476 |
|  |  | T/T | 17（6.3） | 8（7.0） | 1.361 (0.548, 3.382) |  |  |
|  | Log-additive | - | - | - | 1.378 (0.9475, 2.005) | 0.09336 | 0.3792 |
| rs7811989 | Dominant | G/G | 169（62.6） | 60（52.6） | 1 | 0.08781 | 0.3856 |
|  |  | G/A-A/A | 101（37.4） | 54（47.4） | 1.521 (0.9397, 2.463) |  |  |
|  | Recessive | G/G-G/A | 252（93.3） | 106（93.0） | 1 | 0.6154 | 0.7693 |
|  |  | A/A | 18（6.7） | 8（7.0） | 1.26 (0.5119, 3.099) |  |  |
|  | Log-additive | - | - | - | 1.343 (0.9248, 1.95) | 0.1213 | 0.3792 |
| rs2066853 | Dominant | G/G | 111（41.1） | 55（48.2） | 1 | 0.1086 | 0.3856 |
|  |  | G/A-A/A | 159（58.9） | 59（51.8） | 0.6761 (0.4191, 1.091) |  |  |
|  | Recessive | G/G-G/A | 237（87.8） | 105（92.1） | 1 | 0.1271 | 0.5241 |
|  |  | A/A | 33（12.2） | 9（7.9） | 0.5308 (0.2352, 1.198) |  |  |
|  | Log-additive | - | - | - | 0.6974 (0.4834, 1.006) | **0.05394** | **0.3792** |
| rs2040623 | Dominant | A/A | 96（35.6） | 48（42.1） | 1 | 0.1246 | 0.3856 |
|  |  | A/C-C/C | 174（64.4） | 66（57.9） | 0.6815 (0.4178, 1.112) |  |  |
|  | Recessive | A/A-A/C | 228（84.4） | 102（89.5） | 1 | 0.09747 | 0.5241 |
|  |  | CC | 42（15.6） | 12（10.5） | 0.5408 (0.2614, 1.119) |  |  |
|  | Log-additive | - | - | - | 0.701 (0.4906, 1.002) | **0.05111** | **0.3792** |
| rs1048943 | Dominant | T/T | 146（54.1） | 65（57.0） | 1 | 0.5324 | 0.6425 |
|  |  | T/C- C/C | 124（45.9） | 49（43.0） | 0.8599 (0.5355, 1.381) |  |  |
|  | Recessive | T/T- T/C | 254（94.1） | 106（93.0） | 1 | 0.459 | 0.7476 |
|  |  | C/C | 16（5.9） | 8（7.0） | 1.435 (0.5519, 3.73) |  |  |
|  | Log-additive | - | - | - | 0.9562 (0.6477, 1.412) | 0.8216 | 0.8216 |
| rs4646422 | Dominant | C/C | 201（74.4） | 88（77.2） | 1 | 0.4499 | 0.6425 |
|  |  | C/T-T/T | 69（25.6） | 26（22.8） | 0.8068 (0.4623,1.408) |  |  |
|  | Recessive | C/C-C/T | 268（99.3） | 112（98.2） | 1 | 0.4461 | 0.7476 |
|  |  | T/T | 2（0.7） | 2（1.8） | 2.302 (0.2696,19.65) |  |  |
|  | Log-additive | - | - | - | 0.8689 (0.5174,1.459) | 0.5951 | 0.7257 |
| rs4642421 | Dominant | G/G | 84（31.1） | 40（35.1） | 1 | 0.5663 | 0.6425 |
|  |  | G/A-A/A | 186（68.9） | 74（64.9） | 0.8648(0.5263,1.421) |  |  |
|  | Recessive | G/G-G/A | 225（83.3） | 88（77.2） | 1 | 0.131 | 0.5241 |
|  |  | A/A | 45（16.7） | 26（22.8） | 1.578(0.8729,2.854) |  |  |
|  | Log-additive | - | - | - | 1.08 (0.7713,1.513) | 0.6531 | 0.7257 |
| rs762551 | Dominant | A/A | 119（44.1） | 58（50.9） | 1 | 0.1481 | 0.3856 |
|  |  | A/C- C/C | 151（55.9） | 56（49.1） | 0.7065 (0.4413, 1.131) |  |  |
|  | Recessive | A/A- A/C | 235（87.0） | 103（90.4） | 1 | 0.5085 | 0.7476 |
|  |  | C/C | 35（13.0） | 11（9.6） | 0.7747 (0.3635, 1.651) |  |  |
|  | Log-additive | - | - | - | 0.7806 (0.5482, 1.111) | **0.1694** | **0.3792** |
| rs4646425 | Dominant | C/C | 238（88.1） | 98（86.0） | 1 | 0.3443 | 0.6425 |
|  |  | C/T-T/T | 32（11.9） | 16（14.0） | 1.403 (0.6954,2.83) |  |  |
|  | Recessive | C/C-C/T | 270（100.0） | 113（99.1） | 1 | 0.9993 | 0.9993 |
|  |  | T/T | 0（0.0） | 1（0.9） | 3.578e+009(0,inf) |  |  |
|  | Log-additive | - | - | - | 1.481 (0.7574,2.896) | **0.251** | **0.4564** |
| rs2472304 | Dominant | G/G | 189（70.0） | 83（72.8） | 1 | 0.7202 | 0.7202 |
|  |  | G/A-A/A | 81（30.0） | 31（27.2） | 0.9062(0.5288,1.553) |  |  |
|  | Recessive | G/G-G/A | 265（98.1） | 113（99.1） | 1 | 0.5233 | 0.7476 |
|  |  | A/A | 5（1.9） | 1（0.9） | 0.4831(0.05171,4.513) |  |  |
|  | Log-additive | - | - | - | 0.8821 (0.5369,1.449) | 0.6202 | 0.7257 |
| rs2470890 | Dominant | G/G | 189（70.0） | 83（72.8） | 1 | 0.7202 | 0.7202 |
|  |  | G/A-A/A | 81（30.0） | 31（27.2） | 0.9062(0.5288,1.553) |  |  |
|  | Recessive | G/G-G/A | 265（98.1） | 113（99.1） | 1 | 0.5233 | 0.7476 |
|  | Log-additive | A/A  - | 5（1.9）  - | 1（0.9）  - | 0.4831(0.05171,4.513)  0.8821 (0.5369,1.449) |  |  |
|  |  |  |  |  |  | 0.6202 | 0.7257 |
| rs2855658 | Dominant | C/C | 217（80.4） | 100（87.7） | 1 | 0.09394 | 0.3856 |
|  |  | C/T- T/T | 53（19.6） | 14（12.3） | 0.5648 (0.2894, 1.102) |  |  |
|  | Recessive | C/C - C/T | 265（98.1） | 113（99.1） | 1 | 0.7167 | 0.8432 |
|  |  | T/T | 5（1.9） | 1（0.9） | 0.6545 (0.0663, 6.641) |  |  |
|  | Log-additive | - | - | - | 0.6053 (0.3273, 1.119) | 0.1095 | 0.3792 |
| rs1056837 | Dominant | G/G | 217（80.4） | 99（86.8） | 1 | 0.1366 | 0.3856 |
|  |  | G/A-A/A | 53（19.6） | 15（13.2） | 0.6069 (0.3145, 1.171) |  |  |
|  | Recessive | G/G-G/A | 265（98.1） | 113（99.1） | 1 | 0.452 | 0.7476 |
|  |  | A/A | 6（1.9） | 1（0.9） | 0.424 (0.04533, 3.966) |  |  |
|  | Log-additive | - | - | - | 0.6305 (0.3487, 1.14) | 0.127 | 0.3792 |
| rs1056836 | Dominant | G/G | 216（80.0） | 98（86.0） | 1 | 0.1542 | 0.3856 |
|  |  | G/C- C/C | 54（20.0） | 16（14.0） | 0.6266 (0.3294, 1.192) |  |  |
|  | Recessive | G/G - G/C | 264（97.8） | 113（99.1） | 1 | 0.452 | 0.7476 |
|  |  | C/C | 6（2.2） | 1（0.9） | 0.424 (0.04533, 3.966) |  |  |
|  | Log-additive | - | - | - | 0.6463 (0.3616, 1.155) | 0.1408 | 0.3792 |
| rs1056827 | Dominant | C/C | 171（63.3） | 68（59.6） | 1 | 0.363 | 0.6425 |
|  |  | C/A- A/A | 99（36.7） | 46（40.4） | 1.252 (0.7716, 2.03) |  |  |
|  | Recessive | C/C - C/A | 260（96.3） | 108（94.7） | 1 | 0.08369 | 0.5241 |
|  |  | A/A | 10（3.7） | 6（5.3） | 2.625 (0.8794, 7.835) |  |  |
|  | Log-additive | - | - | - | 1.332 (0.8839, 2.007) | 0.1706 | 0.3792 |
| rs10012 | Dominant | G/G | 171（63.3） | 69（60.5） | 1 | 0.4797 | 0.6425 |
|  |  | G/C-C/C | 99（36.7） | 45（39.5） | 1.192 (0.7328,1.938) |  |  |
|  | Recessive | G/G-G/C | 260（96.3） | 108（94.7） | 1 | 0.08369 | 0.5241 |
|  |  | C/C | 10（3.7） | 6（5.3） | 2.625 (0.8794, 7.835) |  |  |
|  | Log-additive | - | - | - | 1.286 (0.8517,1.94) | 0.2318 | 0.4564 |
| rs3813867 | Dominant | G/G | 171（63.3） | 66（57.9） | 1 | 0.4054 | 0.6425 |
|  |  | G/C- C/C | 99（36.7） | 48（42.1） | 1.225 (0.7593, 1.977) |  |  |
|  | Recessive | G/G- G/C | 258（95.6） | 107（93.9） | 1 | 0.8721 | 0.918 |
|  |  | C/C | 12（4.4） | 7（6.1） | 1.087 (0.394, 2.999) |  |  |
|  | Log-additive | - | - | - | 1.157 (0.7852, 1.706) | 0.4602 | 0.708 |
| rs2031920 | Dominant | C/C | 171（63.3） | 66（57.9） | 1 | 0.4054 | 0.6425 |
|  |  | C/T-T/T | 99（36.7） | 48（42.1） | 1.225 (0.7593, 1.977) |  |  |
|  | Recessive | C/C-C/T | 258（95.6） | 107（93.9） | 1 | 0.8721 | 0.918 |
|  |  | T/T | 12（4.4） | 7（6.1） | 1.087 (0.394, 2.999) |  |  |
|  | Log-additive | - | - | - | 1.157 (0.7852, 1.706) | 0.4602 | 0.708 |
| rs915908 | Dominant | G/G | 190（70.4） | 78（68.4） | 1 | 0.5735 | 0.6425 |
|  |  | G/A-A/A | 80（29.6） | 36（31.6） | 1.158(0.6946,1.931) |  |  |
|  | Recessive | G/G-G/A | 258（95.6） | 111（97.4） | 1 | 0.5113 | 0.7476 |
|  |  | A/A | 12（4.4） | 3（2.6） | 0.6257(0.1544,2.536) |  |  |
|  | Log-additive | - | - | - | 1.057 (0.686,1.63) | 0.8004 | 0.8216 |
| rs6413432 | Dominant | T/T | 145（53.7） | 63（55.3） | 1 | 0.5782 | 0.6425 |
|  |  | T/A-A/A | 125（46.3） | 51（44.7） | 0.8738(0.543,1.406) |  |  |
|  | Recessive | T/T-T/A | 248（91.9） | 106（93.0） | 1 | 0.5902 | 0.7693 |
|  |  | A/A | 22（8.1） | 8（7.0） | 0.7836(0.3226,1.903) |  |  |
|  | Log-additive | - | - | - | 0.8819 (0.6078,1.279) | 0.5078 | 0.7254 |

**Table S2-4 Association between maternal genotypes and the risk of left-sided obstructive malformations**

| dbSNP_ID | Model | Genotype | Controls | **left-sided obstructive malformations** | aOR(95%CI) | *P*-value | FDR-BH *P* value |
| --- | --- | --- | --- | --- | --- | --- | --- |
|  |  |  | N(%) | N(%) |  |  |  |
| rs2158041 | Dominant | C/C | 168（62.2） | 43（59.7） | 1 | 0.3964 | 0.6008 |
|  |  | C/T- T/T | 102（37.8） | 29（40.3） | 1.292 ( 0.7148, 2.335) |  |  |
|  | Recessive | C/C- C/T | 253（93.7） | 67（93.1） | 1 | 0.5658 | 0.891 |
|  |  | T/T | 17（6.3） | 5（6.9） | 1.39 (0.4516, 4.279) |  |  |
|  | Log-additive | - | - | - | 1.239 ( 0.7787, 1.972 ) | 0.3657 | 0.5909 |
| rs7811989 | Dominant | G/G | 169（62.6） | 43（59.7） | 1 | 0.3765 | 0.6008 |
|  |  | G/A-A/A | 101（37.4） | 29（40.3） | 1.309 ( 0.7208, 2.377) |  |  |
|  | Recessive | G/G-G/A | 252（93.3） | 67（93.1） | 1 | 0.6683 | 0.891 |
|  |  | A/A | 18（6.7） | 5（6.9） | 1.275 (0.4195, 3.876) |  |  |
|  | Log-additive | - | - | - | 1.228 ( 0.7734, 1.949 ) | 0.3841 | 0.5909 |
| rs2066853 | Dominant | G/G | 111（41.1） | 35（48.6） | 1 | 0.1205 | 0.3416 |
|  |  | G/A-A/A | 159（58.9） | 37（51.4） | 0.6317 (0.3538,1.128) |  |  |
|  | Recessive | G/G-G/A | 237（87.8） | 65（90.3） | 1 | 0.2963 | 0.8465 |
|  |  | A/A | 33（12.2） | 7（9.7） | 0.5981 (0.228,1.569) |  |  |
|  | Log-additive | - | - | - | 0.6899 (0.4433,1.073) | **0.09984** | **0.3516** |
| rs2040623 | Dominant | A/A | 96（35.6） | 32（44.4） | 1 | 0.06565 | 0.3416 |
|  |  | A/C-C/C | 174（64.4） | 40（55.6） | 0.5731 (0.3168, 1.037) |  |  |
|  | Recessive | A/A-A/C | 228（84.4） | 62（86.1） | 1 | 0.6282 | 0.891 |
|  |  | CC | 42（15.6） | 10（13.9） | 0.8153(0.3567, 1.863) |  |  |
|  | Log-additive | - | - | - | 0.7123 (0.4628, 1.096) | **0.123** | **0.3516** |
| rs1048943 | Dominant | T/T | 146（54.1） | 39（54.2） | 1 | 0.9268 | 0.9658 |
|  |  | T/C- C/C | 124（45.9） | 33（45.8） | 1.027( 0.579, 1.823) |  |  |
|  | Recessive | T/T- T/C | 254（94.1） | 67（93.1） | 1 | 0.7551 | 0.9438 |
|  |  | C/C | 16（5.9） | 5（6.9） | 0.8283 (0.2537, 2.705) |  |  |
|  | Log-additive | - | - | - | 0.988 ( 0.6225, 1.568 ) | 0.959 | 0.959 |
| rs4646422 | Dominant | C/C | 201（74.4） | 59（81.9） | 1 | 0.4206 | 0.6008 |
|  |  | C/T-T/T | 69（25.6） | 13（18.1） | 0.7429 (0.3604,1.531) |  |  |
|  | Recessive | C/C-C/T | 268（99.3） | 71（98.6） | 1 | 0.9952 | 0.9989 |
|  |  | T/T | 2（0.7） | 1（1.4） | 1.008 (0.08056,12.61) |  |  |
|  | Log-additive | - | - | - | 0.779 (0.4011,1.513) | 0.4608 | 0.6144 |
| rs4642421 | Dominant | G/G | 84（31.1） | 22（30.6） | 1 | 0.8352 | 0.928 |
|  |  | G/A-A/A | 186（68.9） | 50（69.4） | 1.069 (0.5723, 1.995) |  |  |
|  | Recessive | G/G-G/A | 225（83.3） | 55（76.4） | 1 | 0.6455 | 0.891 |
|  |  | A/A | 45（16.7） | 17（23.6） | 1.185(0.5754, 2.439) |  |  |
|  | Log-additive | - | - | - | 1.086(0.7201, 1.639) | 0.6927 | 0.8149 |
| rs762551 | Dominant | A/A | 119（44.1） | 45（62.5） | 1 | 0.002982 | 0.05964 |
|  |  | A/C- C/C | 151（55.9） | 27（37.5） | **0.4057 ( 0.2236, 0.7358)*** |  |  |
|  | Recessive | A/A- A/C | 235（87.0） | 67（93.1） | 1 | 0.199 | 0.7962 |
|  |  | C/C | 35（13.0） | 5（6.9） | 0.4944 (0.1687, 1.449) |  |  |
|  | Log-additive | - | - | - | **0.5079 ( 0.3157, 0.8172 )*** | **0.005239** | **0.1048** |
| rs4646425 | Dominant | C/C | 238（88.1） | 60（83.3） | 1 | 0.06876 | 0.3416 |
|  |  | C/T-T/T | 32（11.9） | 12（16.7） | 2.139(0.9432,4.85) |  |  |
|  | Recessive | C/C-C/T | 270（100.0） | 70（97.2） | 1 | 0.9989 | 0.9989 |
|  |  | T/T | 0（0.0） | 2（2.8） | 1.007e+010(0,inf) |  |  |
|  | Log-additive | - | - | - | **2.443 (1.161,5.14)*** | **0.01855** | **0.1855** |
| rs2472304 | Dominant | G/G | 189（70.0） | 45（62.5） | 1 | 0.1366 | 0.3416 |
|  |  | G/A-A/A | 81（30.0） | 27（37.5） | 1.594 (0.8626, 2.946) |  |  |
|  | Recessive | G/G-G/A | 265（98.1） | 68（94.4） | 1 | 0.1177 | 0.665 |
|  |  | A/A | 5（1.9） | 4（5.6） | 3.256(0.7419, 14.29) |  |  |
|  | Log-additive | - | - | - | 1.62 (0.9592, 2.735) | 0.07123 | 0.3516 |
| rs2470890 | Dominant | G/G | 189（70.0） | 46（63.9） | 1 | 0.2028 | 0.4507 |
|  |  | G/A-A/A | 81（30.0） | 26（36.1） | 1.495 (0.8053, 2.774) |  |  |
|  | Recessive | G/G-G/A | 265（98.1） | 68（94.4） | 1 | 0.1177 | 0.665 |
|  | Log-additive | A/A  - | 5（1.9）  - | 4（5.6）  - | 3.256(0.7419, 14.29)  1.545 (0.9127, 2.615) |  |  |
|  |  |  |  |  |  | 0.1053 | 0.3516 |
| rs2855658 | Dominant | C/C | 217（80.4） | 50（69.4） | 1 | 0.1222 | 0.3416 |
|  |  | C/T- T/T | 53（19.6） | 22（30.6） | 1.676 ( 0.8708, 3.224) |  |  |
|  | Recessive | C/C - C/T | 265（98.1） | 71（98.6） | 1 | 0.9825 | 0.9989 |
|  |  | T/T | 5（1.9） | 1（1.4） | 0.9735 (0.08817, 10.75) |  |  |
|  | Log-additive | - | - | - | 1.514 ( 0.8438, 2.717 ) | 0.1643 | 0.3727 |
| rs1056837 | Dominant | G/G | 217（80.4） | 50（69.4） | 1 | 0.1009 | 0.3416 |
|  |  | G/A-A/A | 53（19.6） | 22（30.6） | 1.731 (0.8987,3.333) |  |  |
|  | Recessive | G/G-G/A | 265（98.1） | 71（98.6） | 1 | 0.5066 | 0.891 |
|  |  | A/A | 6（1.9） | 1（1.4） | 0.4464 (0.04132,4.823) |  |  |
|  | Log-additive | - | - | - | 1.443 (0.8105,2.57) | 0.2127 | 0.4254 |
| rs1056836 | Dominant | G/G | 216（80.0） | 50（69.4） | 1 | 0.1299 | 0.3416 |
|  |  | G/C- C/C | 54（20.0） | 22（30.6） | 1.656 (0.8621,3.182) |  |  |
|  | Recessive | G/G- G/C | 264（97.8） | 71（98.6） | 1 | 0.5066 | 0.891 |
|  |  | C/C | 6（2.2） | 1（1.4） | 0.4464 (0.04132,4.823) |  |  |
|  | Log-additive | - | - | - | 1.398 (0.7857,2.489) | 0.2543 | 0.4624 |
| rs1056827 | Dominant | C/C | 171（63.3） | 43（59.7） | 1 | 0.3112 | 0.5659 |
|  |  | C/A- A/A | 99（36.7） | 29（40.3） | 1.359 ( 0.7503, 2.463) |  |  |
|  | Recessive | C/C - C/A | 260（96.3） | 68（94.4） | 1 | 0.133 | 0.665 |
|  |  | A/A | 10（3.7） | 4（5.6） | 2.73 (0.7364, 10.12) |  |  |
|  | Log-additive | - | - | - | 1.423 ( 0.8621, 2.349 ) | 0.1677 | 0.3727 |
| rs10012 | Dominant | G/G | 171（63.3） | 43（59.7） | 1 | 0.3112 | 0.5659 |
|  |  | G/C-C/C | 99（36.7） | 29（40.3） | 1.359 ( 0.7503, 2.463) |  |  |
|  | Recessive | G/G-G/C | 260（96.3） | 67（93.1） | 1 | 0.05327 | 0.665 |
|  |  | C/C | 10（3.7） | 5（6.9） | 3.362 (0.983,11.5) |  |  |
|  | Log-additive | - | - | - | 1.474 (0.9007,2.411) | 0.1226 | 0.3516 |
| rs3813867 | Dominant | G/G | 171（63.3） | 43（59.7） | 1 | 0.7424 | 0.8734 |
|  |  | G/C- C/C | 99（36.7） | 29（40.3） | 1.105 ( 0.6104, 1.999) |  |  |
|  | Recessive | G/G- G/C | 258（95.6） | 69（95.8） | 1 | 0.6449 | 0.891 |
|  |  | C/C | 12（4.4） | 3（4.2） | 0.7073 (0.1623, 3.084) |  |  |
|  | Log-additive | - | - | - | 1.028 ( 0.6253, 1.688 ) | 0.9146 | 0.959 |
| rs2031920 | Dominant | C/C | 171（63.3） | 43（59.7） | 1 | 0.7424 | 0.8734 |
|  |  | C/T-T/T | 99（36.7） | 29（40.3） | 1.105 ( 0.6104, 1.999) |  |  |
|  | Recessive | C/C-C/T | 258（95.6） | 69（95.8） | 1 | 0.6449 | 0.891 |
|  |  | T/T | 12（4.4） | 3（4.2） | 0.7073 (0.1623, 3.084) |  |  |
|  | Log-additive | - | - | - | 1.028 ( 0.6253, 1.688 ) | 0.9146 | 0.959 |
| rs915908 | Dominant | G/G | 190（70.4） | 53（73.6） | 1 | 0.9658 | 0.9658 |
|  |  | G/A-A/A | 80（29.6） | 19（26.4） | 0.9857 (0.5114,1.9) |  |  |
|  | Recessive | G/G-G/A | 258（95.6） | 72（100.0） | 1 | 0.9977 | 0.9989 |
|  |  | A/A | 12（4.4） | 0（0.0） | 3.806e-009(0,inf) |  |  |
|  | Log-additive | - | - | - | 0.8664 (0.4797,1.565) | 0.6344 | 0.793 |
| rs6413432 | Dominant | T/T | 145（53.7） | 39（54.2） | 1 | 0.7036 | 0.8734 |
|  |  | T/A-A/A | 125（46.3） | 33（45.8） | 0.8936 (0.5007, 1.595) |  |  |
|  | Recessive | T/T-T/A | 248（91.9） | 68（94.4） | 1 | 0.2891 | 0.8465 |
|  |  | A/A | 22（8.1） | 43（59.7） | 1 |  |  |
|  | Log-additive | - | - | 29（40.3） | 1.292 ( 0.7148, 2.335) | 0.4512 | 0.6144 |

**Table S2-5 Association between maternal genotypes and the risk of anomalous pulmonary venous return**

| dbSNP_ID | Model | Genotype | Controls | **anomalous pulmonary venous return** | aOR(95%CI) | *P*-value | FDR-BH *P* value |
| --- | --- | --- | --- | --- | --- | --- | --- |
|  |  |  | N(%) | N(%) |  |  |  |
| rs2158041 | Dominant | C/C | 168（62.2） | 34（53.1） | 1 | 0.129 | 0.6449 |
|  |  | C/T- T/T | 102（37.8） | 30（46.9） | 1.639 (0.866, 3.103) |  |  |
|  | Recessive | C/C- C/T | 253（93.7） | 56（87.5） | 1 | 0.04248 | 0.582 |
|  |  | T/T | 17（6.3） | 8（12.5） | **2.817 (1.036, 7.661)*** |  |  |
|  | Log-additive | - | - | - | **1.638 (1.019 , 2.631)*** | 0.04152 | 0.2768 |
| rs7811989 | Dominant | G/G | 169（62.6） | 33（51.6） | 1 | 0.08194 | 0.5463 |
|  |  | G/A-A/A | 101（37.4） | 31（48.4） | 1.769 (0.9303, 3.264) |  |  |
|  | Recessive | G/G-G/A | 252（93.3） | 56（87.5） | 1 | 0.06042 | 0.582 |
|  |  | A/A | 18（6.7） | 8（12.5） | 2.576 (0.9594, 6.919) |  |  |
|  | Log-additive | - | - | - | **1.674 (1.044 , 2.684)*** | 0.03232 | 0.2768 |
| rs2066853 | Dominant | G/G | 111（41.1） | 31（48.4） | 1 | 0.2404 | 0.6674 |
|  |  | G/A-A/A | 159（58.9） | 33（51.6） | 0.6874 (0.3677,1.285) |  |  |
|  | Recessive | G/G-G/A | 237（87.8） | 60（93.8） | 1 | 0.1352 | 0.6015 |
|  |  | A/A | 33（12.2） | 4（6.2） | 0.4069 (0.125, 1.324) |  |  |
|  | Log-additive | - | - | - | 0.6704 (0.4113, 1.093) | **0.1087** | **0.5186** |
| rs2040623 | Dominant | A/A | 96（35.6） | 28（43.8） | 1 | 0.2115 | 0.6674 |
|  |  | A/C-C/C | 174（64.4） | 36（56.2） | 0.6664 (0.3525, 1.26) |  |  |
|  | Recessive | A/A-A/C | 228（84.4） | 57（89.1） | 1 | 0.3062 | 0.6852 |
|  |  | CC | 42（15.6） | 7（10.9） | 0.6067 (0.2329, 1.58) |  |  |
|  | Log-additive | - | - | - | 0.7118 (0.4453, 1.138) | **0.1556** | **0.5186** |
| rs1048943 | Dominant | T/T | 146（54.1） | 30（46.9） | 1 | 0.2152 | 0.6674 |
|  |  | T/C- C/C | 124（45.9） | 34（53.1） | 1.482 (0.7956, 2.761) |  |  |
|  | Recessive | T/T- T/C | 254（94.1） | 56（87.5） | 1 | 0.3844 | 0.6988 |
|  |  | C/C | 16（5.9） | 8（12.5） | 1.598（0.5559, 4.592) |  |  |
|  | Log-additive | - | - | - | 1.381 (0.8593, 2.22) | 0.1823 | 0.5208 |
| rs4646422 | Dominant | C/C | 201（74.4） | 53（82.8） | 1 | 0.267 | 0.6674 |
|  |  | C/T-T/T | 69（25.6） | 11（17.2） | 0.64 (0.291,1.407) |  |  |
|  | Recessive | C/C-C/T | 268（99.3） | 63（98.4） | 1 | 0.8978 | 0.9992 |
|  |  | T/T | 2（0.7） | 1（1.6） | 0.8499 (0.07094, 10.18) |  |  |
|  | Log-additive | - | - | - | 0.686 (0.3364,1.399) | 0.2999 | 0.7498 |
| rs4642421 | Dominant | G/G | 84（31.1） | 21（32.8） | 1 | 0.7879 | 0.9991 |
|  |  | G/A-A/A | 186（68.9） | 43（67.2） | 0.9128(0.4698, 1.774) |  |  |
|  | Recessive | G/G-G/A | 225（83.3） | 50（78.1） | 1 | 0.7374 | 0.9992 |
|  |  | A/A | 45（16.7） | 14（21.9） | 1.144 (0.5221, 2.504) |  |  |
|  | Log-additive | - | - | - | 1.002 (0.6415, 1.565) | 0.9932 | 0.9932 |
| rs762551 | Dominant | A/A | 119（44.1） | 36（56.3） | 1 | 0.04302 | 0.4578 |
|  |  | A/C- C/C | 151（55.9） | 28（43.8） | **0.5245 (0.2808, 0.9799)*** |  |  |
|  | Recessive | A/A- A/C | 235（87.0） | 57（89.1） | 1 | 0.9716 | 0.9992 |
|  |  | C/C | 35（13.0） | 7（10.9） | 1.018（0.3895,2.659) |  |  |
|  | Log-additive | - | - | - | 0.6987 (0.4341, 1.125) | **0.1398** | **0.5186** |
| rs4646425 | Dominant | C/C | 238（88.1） | 51（79.7） | 1 | 0.04578 | 0.4578 |
|  |  | C/T-T/T | 32（11.9） | 13（20.3） | **2.354 (1.016, 5.452)*** |  |  |
|  | Recessive | C/C-C/T | 270（100.0） | 63（98.4） | 1 | 0.9992 | 0.9992 |
|  |  | T/T | 0（0.0） | 1（1.6） | 1.169e+010(0,inf) |  |  |
|  | Log-additive | - | - | - | **2.532 (1.145, 5.597)*** | **0.02174** | **0.2768** |
| rs2472304 | Dominant | G/G | 189（70.0） | 44（68.8） | 1 | 0.416 | 0.832 |
|  |  | G/A-A/A | 81（30.0） | 20（31.2） | 1.329 (0.6698, 2.636) |  |  |
|  | Recessive | G/G-G/A | 265（98.1） | 63（98.4） | 1 | 0.937 | 0.9992 |
|  |  | A/A | 5（1.9） | 1（1.6） | 0.9049 (0.0759, 10.79) |  |  |
|  | Log-additive | - | - | - | 1.259 (0.6746, 2.35) | 0.4693 | 0.8532 |
| rs2470890 | Dominant | G/G | 189（70.0） | 44（68.8） | 1 | 0.416 | 0.832 |
|  |  | G/A-A/A | 81（30.0） | 20（31.2） | 1.329 (0.6698, 2.636) |  |  |
|  | Recessive | G/G-G/A | 265（98.1） | 63（98.4） | 1 | 0.937 | 0.9992 |
|  | Log-additive | A/A  - | 5（1.9）  - | 1（1.6）  - | 0.9049 (0.0759, 10.79)  1.259 (0.6746, 2.35) |  |  |
|  |  |  |  |  |  | 0.4693 | 0.8532 |
| rs2855658 | Dominant | C/C | 217（80.4） | 49（76.6） | 1 | 0.8868 | 0.9991 |
|  |  | C/T- T/T | 53（19.6） | 15（23.4） | 1.055 (0.5058, 2.2) |  |  |
|  | Recessive | C/C - C/T | 265（98.1） | 63（98.4） | 1 | 0.8231 | 0.9992 |
|  |  | T/T | 5（1.9） | 1（1.6） | 1.322（0.1147,15.23) |  |  |
|  | Log-additive | - | - | - | 1.065 (0.5495, 2.066) | 0.8518 | 0.9932 |
| rs1056837 | Dominant | G/G | 217（80.4） | 49（76.6） | 1 | 0.8182 | 0.9991 |
|  |  | G/A-A/A | 53（19.6） | 15（23.4） | 1.09 (0.522,2.277) |  |  |
|  | Recessive | G/G-G/A | 265（98.1） | 63（98.4） | 1 | 0.6478 | 0.9967 |
|  |  | A/A | 6（1.9） | 1（1.6） | 0.5697（0.05093,6.371) |  |  |
|  | Log-additive | - | - | - | 1.022 (0.5345,1.952) | 0.5911 | 0.9094 |
| rs1056836 | Dominant | G/G | 216（80.0） | 49（76.6） | 1 | 0.9061 | 0.9991 |
|  |  | G/C- C/C | 54（20.0） | 15（23.4） | 1.045 (0.5017,2.177) |  |  |
|  | Recessive | G/G - G/C | 264（97.8） | 63（98.4） | 1 | 0.6478 | 0.9967 |
|  |  | C/C | 6（2.2） | 1（1.6） | 0.5697（0.05093,6.371) |  |  |
|  | Log-additive | - | - | - | 0.9894 (0.5177,1.891) | 0.9742 | 0.9932 |
| rs1056827 | Dominant | C/C | 171（63.3） | 43（67.2） | 1 | 0.7558 | 0.9991 |
|  |  | C/A- A/A | 99（36.7） | 21（32.8） | 0.9003 (0.4643, 1.745) |  |  |
|  | Recessive | C/C - C/A | 260（96.3） | 61（95.3） | 1 | 0.316 | 0.6852 |
|  |  | A/A | 10（3.7） | 3（4.7） | 2.123（0.4874,9.247) |  |  |
|  | Log-additive | - | - | - | 1.02 (0.5813, 1.79) | 0.9445 | 0.9932 |
| rs10012 | Dominant | G/G | 171（63.3） | 44（68.8） | 1 | 0.6185 | 0.9991 |
|  |  | G/C-C/C | 99（36.7） | 20（31.2） | 0.8431 (0.4308,1.65) |  |  |
|  | Recessive | G/G-G/C | 260（96.3） | 61（95.3） | 1 | 0.316 | 0.6852 |
|  |  | C/C | 10（3.7） | 3（4.7） | 2.123（0.4874,9.247) |  |  |
|  | Log-additive | - | - | - | 0.9747 (0.5515,1.723) | 0.9298 | 0.9932 |
| rs3813867 | Dominant | G/G | 171（63.3） | 39（60.9） | 1 | 0.9686 | 0.9991 |
|  |  | G/C- C/C | 99（36.7） | 25（39.1） | 0.9873 (0.5231, 1.863) |  |  |
|  | Recessive | G/G- G/C | 258（95.6） | 63（98.4） | 1 | 0.1805 | 0.6015 |
|  |  | C/C | 12（4.4） | 1（1.6） | 0.2243 (0.02517 ,1.999) |  |  |
|  | Log-additive | - | - | - | 0.8611 (0.4991 ,1.486) | 0.5911 | 0.9094 |
| rs2031920 | Dominant | C/C | 171（63.3） | 39（60.9） | 1 | 0.9686 | 0.9991 |
|  |  | C/T-T/T | 99（36.7） | 25（39.1） | 0.9873 (0.5231, 1.863) |  |  |
|  | Recessive | C/C-C/T | 258（95.6） | 63（98.4） | 1 | 0.1805 | 0.6015 |
|  |  | T/T | 12（4.4） | 1（1.6） | 0.2243 (0.02517 ,1.999) |  |  |
|  | Log-additive | - | - | - | 0.8611 (0.4991 ,1.486) | 0.5911 | 0.9094 |
| rs915908 | Dominant | G/G | 190（70.4） | 46（71.9） | 1 | 0.9991 | 0.9991 |
|  |  | G/A-A/A | 80（29.6） | 18（28.1） | 1 (0.5,2.001) |  |  |
|  | Recessive | G/G-G/A | 258（95.6） | 59（92.2） | 1 | 0.3426 | 0.6852 |
|  |  | A/A | 12（4.4） | 5（7.8） | 1.893 (0.5068, 7.07) |  |  |
|  | Log-additive | - | - | - | 1.106 (0.6449, 1.898) | 0.7138 | 0.9932 |
| rs6413432 | Dominant | T/T | 145（53.7） | 33（51.6） | 1 | 0.8885 | 0.9991 |
|  |  | T/A-A/A | 125（46.3） | 31（48.4） | 0.9565 (0.5137, 1.781) |  |  |
|  | Recessive | T/T-T/A | 248（91.9） | 62（96.9） | 1 | 0.0873 | 0.582 |
|  |  | A/A | 22（8.1） | 2（3.1） | 0.2506 (0.0513, 1.224) |  |  |
|  | Log-additive | - | - |  |  | 0.3847 | 0.8532 |

**Table S2-6 Association between maternal genotypes and the risk of other cardiac structural abnormalities**

| dbSNP_ID | Model | Genotype | Controls | **other cardiac structural abnormalities** | aOR(95%CI) | *P*-value | FDR-BH *P* value |
| --- | --- | --- | --- | --- | --- | --- | --- |
|  |  |  | N(%) | N(%) |  |  |  |
| rs2158041 | Dominant | C/C | 168（62.2） | 61（59.8） | 1 | 0.689 | 0.9771 |
|  |  | C/T- T/T | 102（37.8） | 41（40.2） | 1.109 (0.669, 1.837) |  |  |
|  | Recessive | C/C- C/T | 253（93.7） | 100（98.0） | 1 | 0.1577 | 0.6222 |
|  |  | T/T | 17（6.3） | 2（2.0） | 0.3334 (0.07264, 1.53) |  |  |
|  | Log-additive | - | - |  | 0.9547 (0.6253, 1.458) | 0.8299 | 0.9221 |
| rs7811989 | Dominant | G/G | 169（62.6） | 61（59.8） | 1 | 0.7263 | 0.9771 |
|  |  | G/A-A/A | 101（37.4） | 41（40.2） | 1.095 (0.6583, 1.822) |  |  |
|  | Recessive | G/G-G/A | 252（93.3） | 100（98.0） | 1 | 0.1281 | 0.6222 |
|  |  | A/A | 18（6.7） | 2（2.0） | 0.3083 (0.06771, 1.404) |  |  |
|  | Log-additive | - | - |  | 0.9331 (0.6113, 1.424) | 0.7484 | 0.8805 |
| rs2066853 | Dominant | G/G | 111（41.1） | 42（41.2） | 1 | 0.9889 | 0.9889 |
|  |  | G/A-A/A | 159（58.9） | 60（58.8） | 1.004 (0.6071, 1.659) |  |  |
|  | Recessive | G/G-G/A | 237（87.8） | 95（93.1） | 1 | 0.084 | 0.6222 |
|  |  | A/A | 33（12.2） | 7（6.9） | 0.4479 (0.1801, 10114) |  |  |
|  | Log-additive | - | - | - | 0.8503 (0.5807, 1.245) | **0.4044** | **0.7325** |
| rs2040623 | Dominant | A/A | 96（35.6） | 35（34.3） | 1 | 0.9283 | 0.9771 |
|  |  | A/C-C/C | 174（64.4） | 67（65.7） | 1.024 (0.606, 1.732) |  |  |
|  | Recessive | A/A-A/C | 228（84.4） | 90（88.2） | 1 | 0.2536 | 0.6341 |
|  |  | CC | 42（15.6） | 12（11.8） | 0.6474 (0.3069, 1.366) |  |  |
|  | Log-additive | - | - | - | 0.9015 (0.6207, 1.309) | **0.586** | **0.7325** |
| rs1048943 | Dominant | T/T | 146（54.1） | 51（50.0） | 1 | 0.3958 | 0.8638 |
|  |  | T/C- C/C | 124（45.9） | 51（50.0） | 1.24 (0.7547, 2.037) |  |  |
|  | Recessive | T/T- T/C | 254（94.1） | 96（94.1） | 1 | 0.9971 | 0.9993 |
|  |  | C/C | 16（5.9） | 6（5.9） | 0.9981 (0.356, 2.798) |  |  |
|  | Log-additive | - | - |  | 1.153 (0.7709, 1.725) | 0.4882 | 0.7325 |
| rs4646422 | Dominant | C/C | 201（74.4） | 82（80.4） | 1 | 0.2071 | 0.7974 |
|  |  | C/T-T/T | 69（25.6） | 20（19.6） | 0.675 (0.3665, 1.243) |  |  |
|  | Recessive | C/C-C/T | 268（99.3） | 100（98.0） | 1 | 0.6692 | 0.9993 |
|  |  | T/T | 2（0.7） | 2（2.0） | 1.565 (0.2005, 12.22) |  |  |
|  | Log-additive | - | - | - | 0.7429 (0.4258, 1.296) | 0.2953 | 0.7325 |
| rs4642421 | Dominant | G/G | 84（31.1） | 30（29.4） | 1 | 0.8887 | 0.9771 |
|  |  | G/A-A/A | 186（68.9） | 72（70.6） | 1.039 (0.6055, 1.784) |  |  |
|  | Recessive | G/G-G/A | 225（83.3） | 85（83.3） | 1 | 0.9992 | 0.9993 |
|  |  | A/A | 45（16.7） | 17（16.7） | 0.9997 (0.5145, 1.943) |  |  |
|  | Log-additive | - | - | - | 1.018 (0.7041, 1.472) | 0.9241 | 0.9727 |
| rs762551 | Dominant | A/A | 119（44.1） | 52（51.0） | 1 | 0.1416 | 0.7974 |
|  |  | A/C- C/C | 151（55.9） | 50（49.0） | 0.6902 (0.4209, 1.132) |  |  |
|  | Recessive | A/A- A/C | 235（87.0） | 96（94.1） | 1 | 0.09785 | 0.6222 |
|  |  | C/C | 35（13.0） | 6（5.9） | 0.4511 (0.1757, 1.158) |  |  |
|  | Log-additive | - | - |  | 0.6898 (0.4685, 1.016) | **0.05991** | **0.5385** |
| rs4646425 | Dominant | C/C | 238（88.1） | 85（83.3） | 1 | 0.07347 | 0.7974 |
|  |  | C/T-T/T | 32（11.9） | 17（16.7） | 1.902 (0.9407, 3.846) |  |  |
|  | Recessive | C/C-C/T | 270（100.0） | 101（99.0） | 1 | 0.9993 | 0.9993 |
|  |  | T/T | 0（0.0） | 1（1.0） | 5.793e+009(0,inf) |  |  |
|  | Log-additive | - | - | - | 1.99 (1.014, 3.906) | **0.0455** | **0.5385** |
| rs2472304 | Dominant | G/G | 189（70.0） | 70（68.6） | 1 | 0.5436 | 0.9771 |
|  |  | G/A-A/A | 81（30.0） | 32（31.4） | 1.182 (0.6885, 2.031) |  |  |
|  | Recessive | G/G-G/A | 265（98.1） | 100（98.0） | 1 | 0.967 | 0.9993 |
|  |  | A/A | 5（1.9） | 2（2.0） | 1.038 (0.1798, 5.987) |  |  |
|  | Log-additive | - | - | - | 1.149 (0.7066, 1.868) | 0.576 | 0.7325 |
| rs2470890 | Dominant | G/G | 189（70.0） | 69（67.6） | 1 | 0.3976 | 0.8638 |
|  |  | G/A-A/A | 81（30.0） | 33（32.4） | 1.262 (0.7364, 2.162) |  |  |
|  | Recessive | G/G-G/A | 265（98.1） | 100（98.0） | 1 | 0.967 | 0.9993 |
|  | Log-additive | A/A  - | 5（1.9）  - | 2（2.0）  - | 1.038 (0.1798, 5.987)  1.211 (0.7465, 1.963) |  |  |
|  |  |  |  |  |  | 0.4386 | 0.7325 |
| rs2855658 | Dominant | C/C | 217（80.4） | 83（81.4） | 1 | 0.7314 | 0.9771 |
|  |  | C/T- T/T | 53（19.6） | 19（18.6） | 0.8964 (0.4799, 1.674) |  |  |
|  | Recessive | C/C - C/T | 265（98.1） | 102（100.0） | 1 | 0.9984 | 0.9993 |
|  |  | T/T | 5（1.9） | 0（0.0） | 2.421e-009(0,inf) |  |  |
|  | Log-additive | - | - |  | 0.8394 (0.4661, 1.512) | 0.5597 | 0.7325 |
| rs1056837 | Dominant | G/G | 217（80.4） | 83（81.4） | 1 | 0.8412 | 0.9771 |
|  |  | G/A-A/A | 53（19.6） | 19（18.6） | 0.9382 (0.5024, 1.752) |  |  |
|  | Recessive | G/G-G/A | 265（98.1） | 102（100.0） | 1 | 0.9982 | 0.9993 |
|  |  | A/A | 6（1.9） | 0（0.0） | 1.459e-009(0,inf) |  |  |
|  | Log-additive | - | - | - | 0.8359 (0.4705, 1.485) | 0.541 | 0.7325 |
| rs1056836 | Dominant | G/G | 216（80.0） | 82（80.4） | 1 | 0.8691 | 0.9771 |
|  |  | G/C- C/C | 54（20.0） | 20（19.6） | 0.9496 (0.5134, 1.756) |  |  |
|  | Recessive | G/G - G/C | 264（97.8） | 102（100.0） | 1 | 0.9982 | 0.9993 |
|  |  | C/C | 6（2.2） | 0（0.0） | 1.459e-009(0,inf) |  |  |
|  | Log-additive | - | - | - | 0.8466 (0.4801, 1.493) | 0.5649 | 0.7325 |
| rs1056827 | Dominant | C/C | 171（63.3） | 59（57.8） | 1 | 0.07977 | 0.7974 |
|  |  | C/A- A/A | 99（36.7） | 43（42.2） | 1.579 (0.9473, 2.631) |  |  |
|  | Recessive | C/C - C/A | 260（96.3） | 101（99.0） | 1 | 0.4535 | 0.9069 |
|  |  | A/A | 10（3.7） | 1（1.0） | 0.4463 (0.05417, 3.678) |  |  |
|  | Log-additive | - | - |  | 1.372 (0.8719, 2.159) | 0.1715 | 0.7325 |
| rs10012 | Dominant | G/G | 171（63.3） | 62（60.8） | 1 | 0.2392 | 0.7974 |
|  |  | G/C-C/C | 99（36.7） | 40（39.2） | 1.361 (0.8147, 2.273) |  |  |
|  | Recessive | G/G-G/C | 260（96.3） | 101（99.0） | 1 | 0.4535 | 0.9069 |
|  |  | C/C | 10（3.7） | 1（1.0） | 0.4463 (0.05417, 3.678) |  |  |
|  | Log-additive | - | - | - | 1.218 (0.7704, 1.924) | 0.3992 | 0.7325 |
| rs3813867 | Dominant | G/G | 171（63.3） | 69（67.6） | 1 | 0.4319 | 0.8638 |
|  |  | G/C- C/C | 99（36.7） | 33（32.4） | 0.8112 (0.4815, 1.367) |  |  |
|  | Recessive | G/G- G/C | 258（95.6） | 99（97.1） | 1 | 0.2178 | 0.6222 |
|  |  | C/C | 12（4.4） | 3（2.9） | 0.4198 (0.1056, 1.669) |  |  |
|  | Log-additive | - | - |  | 0.7797 (0.5017, 1.212) | 0.2689 | 0.7325 |
| rs2031920 | Dominant | C/C | 171（63.3） | 69（67.6） | 1 | 0.4319 | 0.8638 |
|  |  | C/T-T/T | 99（36.7） | 33（32.4） | 0.8112 (0.4815, 1.367) |  |  |
|  | Recessive | C/C-C/T | 258（95.6） | 99（97.1） | 1 | 0.2178 | 0.6222 |
|  |  | T/T | 12（4.4） | 3（2.9） | 0.4198 (0.1056, 1.669) |  |  |
|  | Log-additive | - | - | - | 0.7797 (0.5017, 1.212) | 0.2689 | 0.7325 |
| rs915908 | Dominant | G/G | 190（70.4） | 74（72.5） | 1 | 0.7821 | 0.9771 |
|  |  | G/A-A/A | 80（29.6） | 28（27.5） | 0.9246 (0.5304, 1.612) |  |  |
|  | Recessive | G/G-G/A | 258（95.6） | 96（94.1） | 1 | 0.5731 | 0.9993 |
|  |  | A/A | 12（4.4） | 6（5.9） | 1.398 (0.4359, 4.484) |  |  |
|  | Log-additive | - | - | - | 0.9962 (0.6365, 1.559) | 0.9866 | 0.9866 |
| rs6413432 | Dominant | T/T | 145（53.7） | 62（60.8） | 1 | 0.1612 | 0.7974 |
|  |  | T/A-A/A | 125（46.3） | 40（39.2） | 0.6968 (0.4204, 1.155) |  |  |
|  | Recessive | T/T-T/A | 248（91.9） | 97（95.1） | 1 | 0.124 | 0.6222 |
|  |  | A/A | 22（8.1） | 5（4.9） | 0.6968 (0.4204, 1.155) |  |  |
|  | Log-additive | - | - | - | 0.6974 (0.4654, 1.045) | 0.08078 | 0.5385 |

aOR: adjusted odds ration, adjusted for maternal age, gestational week, housing renovation, factory or landfill nearby, cooking at home, parental smoking or ETS exposure, maternal drinking, folic acid supplements.

| **Appendix C:**  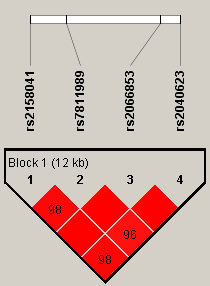 | 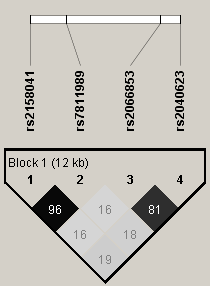 | | 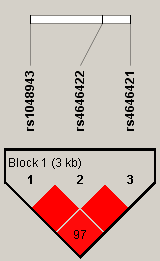 | 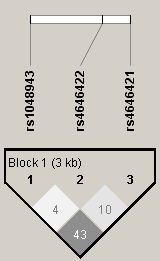 | 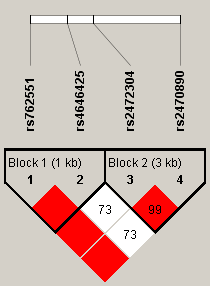 | | 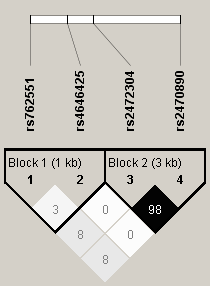 |
| --- | --- | --- | --- | --- | --- | --- | --- |
| AHR: D’ | R^2^ | | CYP1A1: D’ | R^2^ | CYP1A2: D’ | | R^2^ |
| 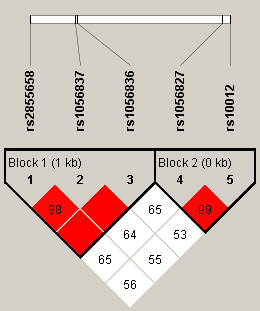 | | 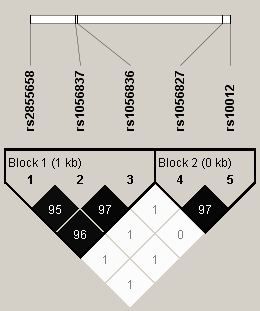 | | 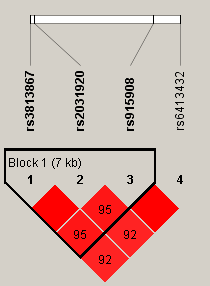 | | 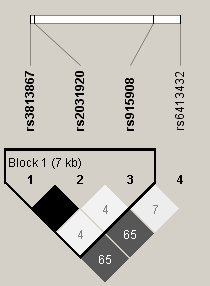 | |
| CYP1B1: D’ | | R^2^ | | CYP2E1: D’ | | R^2^ | |

**Fig S1: Linkage Disequilibrium Analysis of SNPs**

**Appendix D**

**Determination of 1-OHPG concentration**

The deuterated internal standard 1-OHP-d_9_-G was added to 1.0 mL of urine sample at concentration of 2.5 ng/mL, and then 2.0 mL of [ethylacetate](../../../../C:/Documents%20and%20Settings/HP/Local%20Settings/Application%20Data/Yodao/DeskDict/frame/20160116155240/javascript:void(0)%3B) was added to the urine sample and mixed with vortex vibration for 1min. The phase seperation was done by centrifugation at 10000 rpm for 2 min. Then 0.2 mL of [aqueous](../../../../C:/Documents%20and%20Settings/HP/Local%20Settings/Application%20Data/Yodao/DeskDict/frame/20160116155240/javascript:void(0)%3B) [phase](../../../../C:/Documents%20and%20Settings/HP/Local%20Settings/Application%20Data/Yodao/DeskDict/frame/20160116155240/javascript:void(0)%3B) was removed and diluted to 1.0 mL with 5 mmol/L [ammonium](../../../../C:/Documents%20and%20Settings/HP/Local%20Settings/Application%20Data/Yodao/DeskDict/frame/20160116155240/javascript:void(0)%3B) [acetate](../../../../C:/Documents%20and%20Settings/HP/Local%20Settings/Application%20Data/Yodao/DeskDict/frame/20160116155240/javascript:void(0)%3B), and 5 μl of aliquot was injected into the UPLC-MS/MS system after filtration with 0.22μm membrane.

The chromatographic separation was carried out on a Hypersil Gold C_18_ column (50mm×2.1mm i.d., 1.9μm, Thermo Fisher Scientific) with a Accucore XL C_18_ guard cartridge (10×4mm id., 4μm, Thermo Fisher Scientific) maintained at 40 °C. The mobile phase was composed of methanol (eluent A) and water containing 5mmol/L [ammonium](../../../../C:/Documents%20and%20Settings/HP/Local%20Settings/Application%20Data/Yodao/DeskDict/frame/20160116155240/javascript:void(0)%3B) [acetate](../../../../C:/Documents%20and%20Settings/HP/Local%20Settings/Application%20Data/Yodao/DeskDict/frame/20160116155240/javascript:void(0)%3B) (eluent B) at a flow rate of 0.2mL/min. The gradient elution condition was following: 0min-10min，90% B-10% B，equilibrated by 90% B for 2min. The sample injection volume was 5 μl.

The MS/MS ion source operating conditions were set as follows: collision gas (Argon) at 1.5m Torr, spray voltage at -2500 V, sheath gas, auxiliary gas and ion sweep gas (Nitrogen) at 35 Arb, 10 Arb and 2.0 Arb respectively, capillary temperature and vaporizer temperature were both 350 ℃. Quantitative analyses were performed using selected reaction monitoring (SRM) mode. Table1 shows the optimal SRM conditions..

**Table S3 ptimal SRM parameters for determination of 1-OHP-G and 1-OHP-d_9_**-G

| Compounds | Precursor Ions, m/z | Product Ions, m/z | Collision Energy, V | S-Lens |
| --- | --- | --- | --- | --- |
| 1-OHP-G | 393 | 217* | 42 | 99 |
| 1-OHP-G | 393 | 175 | 15 | 99 |
| 1-OHP-G | 393 | 113 | 17 | 99 |
| 1-OHP-d_9_-G | 402 | 226* | 39 | 76 |
| 1-OHP-d_9_-G | 402 | 175 | 15 | 76 |
| 1-OHP-d_9_-G | 402 | 113 | 17 | 76 |

* mean the quantitive ion

The deuterated internal standard 1-OHP-d_9_-G was added to 1.0 mL of urine sample at concentration of 2.5 ng/mL, and then 2.0 mL of [ethylacetate](../../../../C:/Documents%20and%20Settings/HP/Local%20Settings/Application%20Data/Yodao/DeskDict/frame/20160116155240/javascript:void(0)%3B) was added to the urine sample and mixed with vortex vibration for 1min. The phase seperation was done by centrifugation at 10000 rpm for 2 min. Then 0.2 mL of [aqueous](../../../../C:/Documents%20and%20Settings/HP/Local%20Settings/Application%20Data/Yodao/DeskDict/frame/20160116155240/javascript:void(0)%3B) [phase](../../../../C:/Documents%20and%20Settings/HP/Local%20Settings/Application%20Data/Yodao/DeskDict/frame/20160116155240/javascript:void(0)%3B) was removed and diluted to 1.0 mL with 5 mmol/L [ammonium](../../../../C:/Documents%20and%20Settings/HP/Local%20Settings/Application%20Data/Yodao/DeskDict/frame/20160116155240/javascript:void(0)%3B) [acetate](../../../../C:/Documents%20and%20Settings/HP/Local%20Settings/Application%20Data/Yodao/DeskDict/frame/20160116155240/javascript:void(0)%3B), and 5 μl of aliquot was injected into the UPLC-MS/MS system after filtration with 0.22μm membrane.

**Appendix E:**


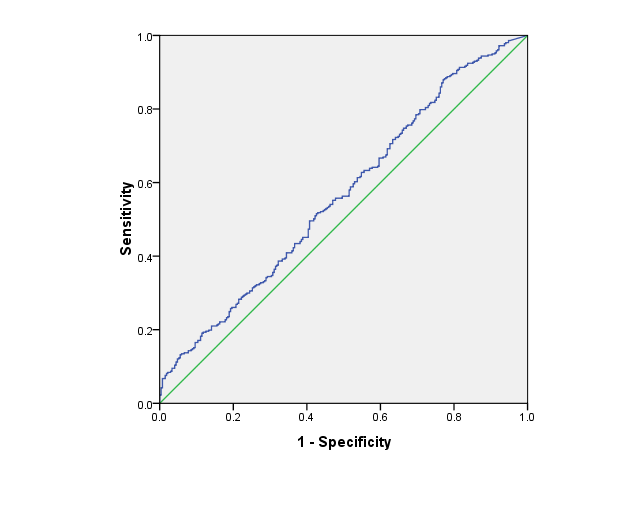
Figure S2. Receiver operating characteristic curve

| **Table S4. Area Under the Curve** | | | | |
| --- | --- | --- | --- | --- |
| Area | Std. Error^a^ | Asymptotic Sig.^b^ | Asymptotic 95% Confidence Interval | |
|  |  |  | Lower Bound | Upper Bound |
| .564 | .023 | .006 | .519 | .609 |
| The test result variable(s): 1-OHPG Concentration has at least one tie between the positive actual state group and the negative actual state group. Statistics may be biased. | | | | |
| a. Under the nonparametric assumption | | | |  |
| b. Null hypothesis: true area = 0.5 | | |  |  |

**Appendix F:**

**Table S5 The information of SNPs**

| **Gene** | **dbSNP_ID** | **Allele** | **chr** | **Chr. Position** | **Ref mRNA** | **SNP Property** | **Functional Change** | **HapMap-HCB** |
| --- | --- | --- | --- | --- | --- | --- | --- | --- |
| AHR | rs2158041 | C/T | 7 | 17368420 | NM_001621.4 | intron4 | / | 0.256 |
| AHR | rs7811989 | G/A | 7 | 17371363 | NM_001621.4 | intron6 | / | 0.232 |
| AHR | rs2066853 | G/A | 7 | 17379110 | NM_001621.4 | nonsynon_exon10 | p.Arg554Lys | 0.394 |
| AHR | rs2040623 | C/A | 7 | 17380662 | NM_001621.4 | intron10 | / | 0.416 |
| CYP1A1 | rs1048943 | C/T | 15 | 75012985 | NM_000499.3 | nonsynon_exon7 | p.Ile462Val | 0.254 |
| CYP1A1 | rs4646422 | C/T | 15 | 75015305 | NM_000499.3 | nonsynon_exon2 | p.Gly45Asp | 0.124 |
| CYP1A1 | rs4646421 | G/A | 15 | 75016192 | NM_000499.3 | intron1 | / | 0.398 |
| CYP1A2 | rs762551 | C/A | 15 | 75041917 | NM_000761.3 | intron1 | / | 0.332 |
| CYP1A2 | rs4646425 | C/T | 15 | 75043281 | NM_000761.3 | intron2 | / | 0.095 |
| CYP1A2 | rs2472304 | G/A | 15 | 75044238 | NM_000761.3 | intron4 | / | 0.157 |
| CYP1A2 | rs2470890 | C/T | 15 | 75047426 | NM_000761.3 | synon_exon7 | p.=(Asn516Asn) | 0.161 |
| CYP1B1 | rs2855658 | C/T | 2 | 38296890 | NM_000104.3 | 3'-UTR | / | 0.096 |
| CYP1B1 | rs1056837 | G/A | 2 | 38298150 | NM_000104.3 | synon_exon3 | p.（Asp449Asp） | 0.093 |
| CYP1B1 | rs1056836 | G/C | 2 | 38298203 | NM_000104.3 | nonsynon_exon3 | p.Leu432Val | 0.095 |
| CYP1B1 | rs1056827 | C/A | 2 | 38302177 | NM_000104.3 | nonsynon_exon2 | p.Ala119Ser | 0.125 |
| CYP1B1 | rs10012 | G/C | 2 | 38302390 | NM_000104.3 | nonsynon_exon2 | p.Arg48Gly | 0.23317 |
| CYP2E1 | rs3813867 | G/C | 10 | 135339605 | NM_000773.3 | 5'-flanking | / | 0.289 |
| CYP2E1 | rs2031920 | C/T | 10 | 135339845 | NM_000773.3 | 5'-flanking | / | 0.252 |
| CYP2E1 | rs915908 | G/A | 10 | 135346959 | NM_000773.3 | intron5 | / | 0.15 |
| CYP2E1 | rs6413432 | A/T | 10 | 135348544 | NM_000773.3 | intron6 | / | 0.26923 |
